# A comparative analysis of RNA-Seq and NanoString technologies in deciphering viral infection response in upper airway lung organoids

**DOI:** 10.3389/fgene.2024.1327984

**Published:** 2024-06-18

**Authors:** Mostafa Rezapour, Stephen J. Walker, David A. Ornelles, Muhammad Khalid Khan Niazi, Patrick M. McNutt, Anthony Atala, Metin Nafi Gurcan

**Affiliations:** ^1^ Center for Artificial Intelligence Research, Wake Forest University School of Medicine, Winston-Salem, NC, United States; ^2^ Wake Forest Institute for Regenerative Medicine, Wake Forest University School of Medicine, Winston-Salem, NC, United States; ^3^ Department of Microbiology and Immunology, Wake Forest University School of Medicine, Winston-Salem, NC, United States

**Keywords:** RNA-Seq, NanoString technologies, infected lung organoids, gene expression analysis, concordance analysis

## Abstract

In this study, we delved into the comparative analysis of gene expression data across RNA-Seq and NanoString platforms. While RNA-Seq covered 19,671 genes and NanoString targeted 773 genes associated with immune responses to viruses, our primary focus was on the 754 genes found in both platforms. Our experiment involved 16 different infection conditions, with samples derived from 3D airway organ-tissue equivalents subjected to three virus types, influenza A virus (IAV), human metapneumovirus (MPV), and parainfluenza virus 3 (PIV3). Post-infection measurements, after UV (inactive virus) and Non-UV (active virus) treatments, were recorded at 24-h and 72-h intervals. Including untreated and Mock-infected OTEs as control groups enabled differentiating changes induced by the virus from those arising due to procedural elements. Through a series of methodological approaches (including Spearman correlation, Distance correlation, Bland-Altman analysis, Generalized Linear Models Huber regression, the Magnitude-Altitude Score (MAS) algorithm and Gene Ontology analysis) the study meticulously contrasted RNA-Seq and NanoString datasets. The Magnitude-Altitude Score algorithm, which integrates both the amplitude of gene expression changes (magnitude) and their statistical relevance (altitude), offers a comprehensive tool for prioritizing genes based on their differential expression profiles in specific viral infection conditions. We observed a strong congruence between the platforms, especially in identifying key antiviral defense genes. Both platforms consistently highlighted genes including ISG15, MX1, RSAD2, and members of the OAS family (OAS1, OAS2, OAS3). The IFIT proteins (IFIT1, IFIT2, IFIT3) were emphasized for their crucial role in counteracting viral replication by both platforms. Additionally, CXCL10 and CXCL11 were pinpointed, shedding light on the organ tissue equivalent’s innate immune response to viral infections. While both platforms provided invaluable insights into the genetic landscape of organoids under viral infection, the NanoString platform often presented a more detailed picture in situations where RNA-Seq signals were more subtle. The combined data from both platforms emphasize their joint value in advancing our understanding of viral impacts on lung organoids.

## 1 Introduction

The human airways are a nexus of intricate relationships, intricately binding cellular interactions, extracellular matrix (ECM) proteins, and the biomechanical milieu. At the forefront of replicating these complexities, our group has innovated a 3D airway organ tissue equivalent (OTE) model functioning at an air-liquid interface (ALI). Incorporating native pulmonary fibroblasts, solubilized lung ECM, and a tunable hydrogel substrate, this model stands as a revolutionary contribution to airway biology research. By evaluating the influence of our model on the phenotype of human bronchial epithelial (HBE) cells over a 28-day ALI culture duration, we’ve noted its pronounced ability in nurturing well-differentiated ALI cultures. These cultures notably manifest barrier functionality and mature epithelial marker expression. A unique feature of our model is the adjustable stiffness of the hydrogel, offering potential avenues for further phenotype modulation research. This paper builds on foundational methodologies previously laid down by our group, emphasizing the versatility and precision of the 3D airway OTE model in simulating the multifarious dimensions of the human airway’s 3D microenvironment (Leach et al., 2023).

To enhance our understanding of the 3D airway OTE model’s response to viral infections, we employed RNA-Seq (Wang et al., 2009) and NanoString (Geiss et al., 2008) technologies to dissect the complex virus-host interactions at the molecular level. We focused on samples collected at two critical time points, 24- and 72-h post-infection, to capture both the immediate and prolonged cellular responses to viral invasion. This strategy aims to provide a comprehensive view of the dynamic interactions between host cells and infecting viruses during these pivotal infection phases.

In a recent study (Rezapour et al., 2024), we analyzed RNA-Seq data encompassing 19,671 genes to explore gene expression dynamics following infection with both active and UV-inactivated viruses: Influenza A virus (IAV), Human metapneumovirus (MPV), and Parainfluenza virus type 3 (PIV3). We employed two algorithms, GLMQL-MAS and GLMQL-Relaxed-MAS, which integrate Generalized Linear Models (GLM), Quasi-Likelihood (QL) F-tests, and the Magnitude-Altitude Score (MAS). These methodologies robustly identified key differentially expressed genes, particularly those involved in interferon signaling pathways such as IFIT1, IFIT2, IFIT3, and OAS1, which play crucial roles in the innate immune response.

This paper undertakes a comparative analysis to demonstrate the consistency of gene selection between the RNA-Seq and NanoString platforms, with the aim of validating the reproducibility of gene expression data across these technologies. Our objectives are as follows:

1. Correlation analysis: We assess the consistency between RNA-Seq and NanoString data across multiple infection conditions using Spearman and Distance correlation metrics, providing a comprehensive evaluation of the agreement between these two platforms.
2. Bland-Altman analysis: This analysis aids in visualizing the level of concordance between RNA-Seq and NanoString measurements, highlighting any systematic biases or discrepancies to enhance our understanding of each platform’s reliability.
3. Generalized linear model and huber regression analysis: By employing these robust statistical tools, we aim to evaluate the relationship between RNA-Seq and NanoString data across diverse infection conditions, ensuring that our findings are resilient against potential data outliers and deviations.
4. Concordance analysis: Utilizing expression analysis and the GLMQL-MAS algorithm (Rezapour et al., 2024), we identify biologically meaningful changes in gene expression, ensuring that the significant genes detected remain consistent across both technological platforms.
5. Gene ontology (GO) analysis for common BH-significant transcripts across two platforms: This analysis confirms the biological relevance of the significant changes detected by both platforms, enriching our understanding of the molecular mechanisms underlying the response to viral infections.

**FIGURE 1:**
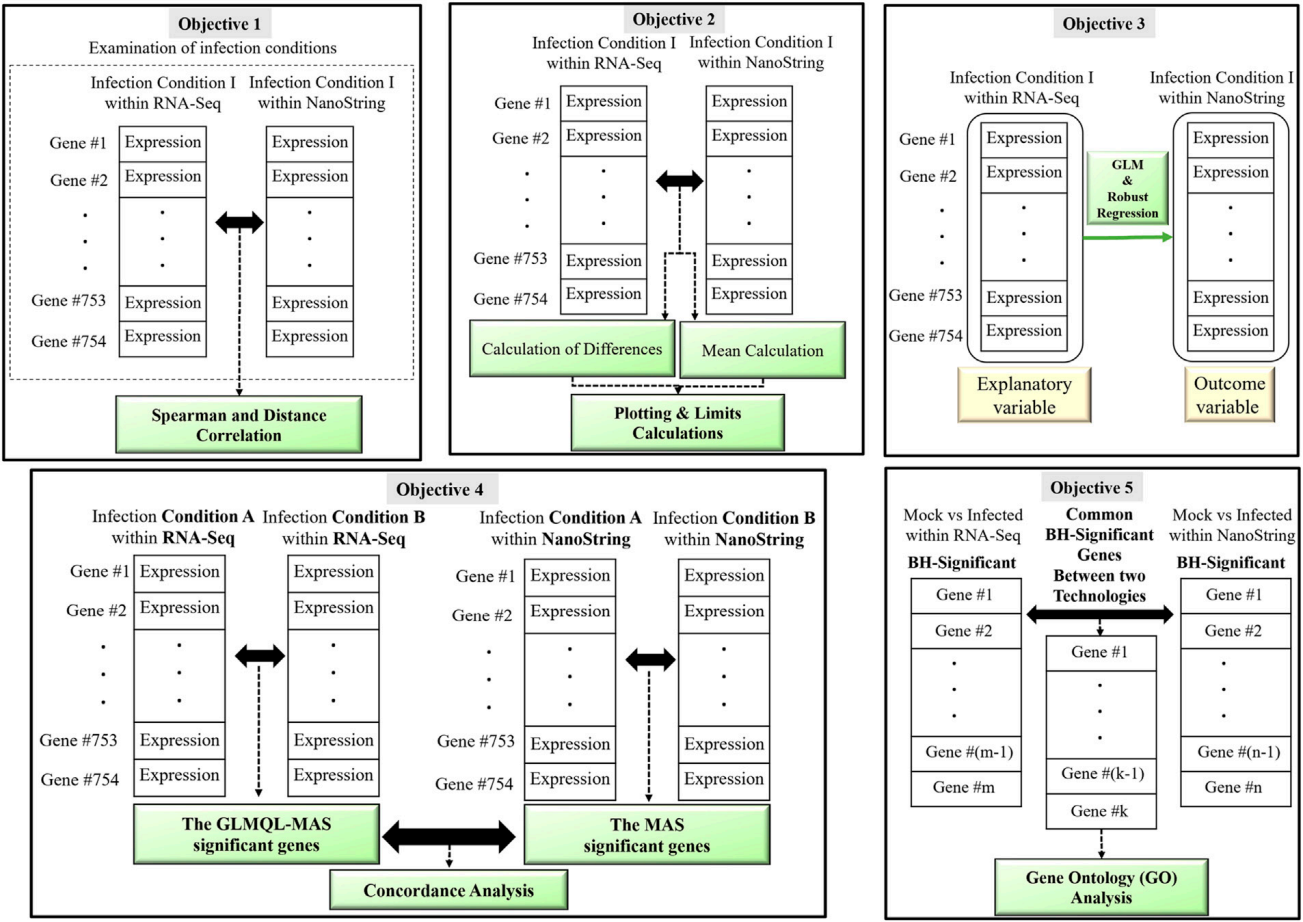
A visual representation illustrating the main objectives of the comparative analysis between RNA-Seq and NanoString platforms. The figure highlights the five key objectives: 1) Correlation Analysis, 2) Bland-Altman Analysis, 3) Regression and Residual Analysis, 4) Concordance Analysis and 5) Gene Ontology Analysis.

Figure 1 displays a schematic overview of the main objectives of our comparative analysis between RNA-Seq and NanoString platforms. By addressing these objectives, our study not only aims to validate the agreement between RNA-Seq and NanoString technologies but also to enhance the biological insights derived from the 3D airway OTE model.

## 2 Materials and methods

In this study, we explored the 3D airway OTE model’s interaction with three distinct viruses:

• Influenza A Virus (IAV): A/Puerto Rico/8/1934 (H1N1) strain, featuring an EGFP-NS1 gene fusion, provided by Adolfo Garcia-Sastre at the Mount Sinai School of Medicine.
• Human Metapneumovirus (MPV): Strain CAN97-83, with EGFP gene upstream of the N gene (ViraTree product #M121).
• Human Parainfluenza Virus Type III (PIV3): Strain JS, incorporating the EGFP gene between the initial (N) and subsequent (P/C/D/V) genes (ViraTree product #P323).

The virus infection medium (iDMEM) was concocted using Dulbecco-modified Eagle’s minimal essential medium, supplemented with 0.1% heat-inactivated fetal bovine serum, 0.3% purified bovine serum albumin, 20 mM HEPES [pH 7.5], and 0.2 mM Glutamax. The concoction was subsequently filter-sterilized through a 0.2 µm filter and preserved at 4°C. Titers of the Influenza virus were ascertained using Madin-Darby Canine Kidney (MDCK) cells (ATCC, #CCL-34), while the titers for all other viruses were determined using the LLC-MK2 rhesus monkey kidney cell line (ATCC, #CCL-185). Each lung OTE was calculated to encompass approximately 1.6 × 10^5^ epithelial cells. The nominal multiplicity of infection was calculated based on the premise that solely the epithelial cells were vulnerable to the viral infections. A 24-well culture plate filled with modified PneumaCult ALI Medium (Stemcell Technologies) was permitted to reach equilibrium at 37°C in a humidified incubator with 5% CO_2_ for 30 min. Inserts holding the OTEs were sterily relocated to the balanced culture plate. The surface of each OTE slated for infection or Mock infection was rinsed by delicately adding 0.3 mL of warm Hank’s balanced saline solution, followed by cautious aspiration. The virus, diluted in iDMEM and brought to ambient temperature, was then dispensed in a 40 µL volume onto the apical surface of the OTE. Subsequently, the plate harboring the infected OTEs was situated on a rocking table in the 37°C CO_2_ incubator for 1 hour before being retrieved from the rocking table and restored to standard growth conditions for the designated durations.

The RNA extraction from the OTEs was meticulously performed using the Direct-zol RNA Miniprep Plus Kit. This protocol included sample preparation, cell lysis, RNA purification, and DNase I treatment to eliminate potential DNA contaminants. We stored the purified RNA at −80°C to preserve it for subsequent in-depth sequencing and gene expression analyses. These steps were critical for accurately dissecting the complex interplay between the OTE model and the introduced viral pathogens, ensuring the integrity of the samples for further molecular analysis.

Following RNA extraction, we conducted RNA sequencing to delve deeper into the molecular underpinnings of virus-host interactions. We crafted cDNA libraries from 50 ng of the extracted RNA using the NEXTFLEX^®^ Combo-Seq™ mRNA/miRNA Kit. The processing was performed on a Sciclone^®^ G3 NGSx Workstation, with libraries quantified using a KAPA Library Quantification Kit and evaluated for average fragment size using a 4,200 TapeStation System. After library normalization, we performed high-throughput sequencing on an Illumina^®^ NovaSeq 6000 System, producing 76-bp single-end reads. This setup laid the groundwork for comprehensive sequence analysis.

For data analysis, we utilized the Partek^®^ Flow^®^ software. The raw sequence data underwent meticulous processing that included adaptor trimming and quality base filtering with Cutadapt (Martin, 2011), alignment against the hg38 GENCODE reference database using the STAR algorithm, and transcript quantification using an expectation/maximization (E/M) algorithm (Xing et al., 2006). We normalized transcript-level counts to gene-level data employing the median-of-ratios method from DESeq2 (Love et al., 2014), with results log2-transformed for enhanced clarity.

Additionally, we leveraged the NanoString nCounter^®^ Analysis System to perform highly multiplexed detection of mRNA targets relevant to our study of viral infections. This technology is particularly suited for samples like those derived from our OTE model where RNA integrity is variable, as it does not rely on amplification or fluorescence intensity for target detection. Instead, detection is based on barcoded sample processing, which yields precise and reproducible gene expression data. We ensured data quality at every analysis stage, beginning with general assay performance and followed by background correction, data normalization, and comprehensive quality control checks on the resultant expression metrics.

Two distinct normalization methods were applied to the RNA-Seq and NanoString data. The RNA-Seq data were normalized using the Trimmed Mean of M-values (TMM) method. This method scales the library sizes by a normalization factor, thus allowing for more accurate comparisons by mitigating the influence of highly expressed genes. For the NanoString data, a two-step normalization process was used. Firstly, a Positive Control Normalization factor was calculated using the positive controls added to each sample. This step helps to adjust for technical variations across samples, lanes, cartridges, and different days of experimentation. Secondly, a CodeSet Content Normalization factor was computed using housekeeping genes.

In our study, special emphasis is placed on Non-UV (active) samples, where active viral infections are facilitated, allowing the viruses to replicate and dynamically interact with the host cells within the OTEs. This condition is pivotal as it most accurately simulates the natural infection environment, providing critical insights into the host’s cellular and molecular responses under active viral attack. To ensure the accuracy of our findings and clearly delineate the effects of viral infections, our study utilized a robust set of control conditions, including Mock, UV-treated, and naïve (untreated) samples. UV-treated samples, exposed to ultraviolet light to inactivate the viruses, served as crucial controls for examining the impact of viral components without active replication. Naïve samples, which are untreated OTEs, acted as internal controls to set a baseline for gene expression across our experiments. Mock-infected samples, treated with a vehicle or sham procedure, offered comparative data to underscore the specific gene expression responses triggered by active viral infections in the Non-UV (active) samples, where viruses capable of replication were used.

Our analyses covered 773 immune response genes identified in the NanoString dataset and a broader spectrum of 19,671 genes covered by RNA-Seq. These genes were evaluated across 16 distinct infection conditions, each involving six replicates of OTEs, as detailed in Supplementary Table S1 (where “S” stands for Supplementary Material). The conditions were categorized by virus type (IAV, MPV, or PIV3), treatment type (UV or Non-UV/active/None), and post-infection times (24-h and 72-h post infection), which is denoted as Virus-Treatment-Time. This comprehensive setup allowed us to rigorously test and validate the biological significance and reproducibility of our data across different experimental and control conditions, as detailed in Supplementary Table S2, which illustrates the distribution of data across these groups. From the available data, we identified 754 genes that were common to both the RNA-Seq and NanoString platforms, facilitating a consistent and comparative analysis of gene expression dynamics in response to viral challenges, particularly focusing on the critical role of Non-UV (active) samples in our study.

### 2.1 Correlation analysis

In this section, we outline the correlation-based methodology adopted to assess the congruence between gene expression data obtained from the RNA-Seq and NanoString platforms across 16 diverse infection conditions. Our approach leveraged two correlation-based metrics: Spearman correlation (Hauke and Kossowski, 2011), and Distance correlation (Székely and Rizzo, 2009) with the aim of providing a comprehensive analysis of the consistency between the two platforms.

Spearman’s correlation is advantageous as it captures not only linear relationships but also non-linear associations. This is particularly useful when gene expression values do not follow a strict linear pattern. Additionally, Spearman’s correlation is less sensitive to outliers, making it a robust choice when dealing with potential noise or extreme values in gene expression data. By assessing the rank-order relationships between gene expression profiles, Spearman’s correlation can provide a holistic view of the agreement between RNA-Seq and NanoString data without being overly influenced by individual data points.

Distance correlation (Székely and Rizzo, 2009) is a measure of statistical dependence that quantifies both linear and non-linear relationships between variables. It offers an advantage in assessing agreement between gene expression profiles of two platforms by accounting for complex associations that may not be adequately captured by linear methods. Distance correlation can detect non-linear patterns and is applicable to high-dimensional data, which makes it suitable for comparing gene expression profiles.

Examination of infection conditions: We delved into the examination of individual infection conditions to examine the variation in gene expression data between RNA-Seq and NanoString technologies within each condition. For this purpose, we created 754-dimensional vectors encompassing the gene expression profiles of all common genes associated with a particular infection condition on both platforms. Spearman and Distance correlation coefficients were chosen as our analytical tools. The selection of these measures was driven by their ability to comprehensively capture various aspects of the relationship between gene expression profiles, facilitating a thorough examination of agreement while considering both parametric and non-parametric aspects of the data (see Figure 1 (objective 1)).

### 2.2 Bland-Altman analysis

Bland-Altman analysis (Giavarina, 2015) is an appropriate technique to assess the agreement between RNA-Seq and NanoString platforms because it provides a visual and statistical approach to evaluating the level of agreement and potential biases between two measurement methods. This method allows us to assess the degree of agreement by plotting the differences between paired measurements against their means, revealing any systematic biases or trends. By identifying potential patterns in the differences and calculating metrics such as mean difference and limits of agreement, Bland-Altman analysis offers valuable insights into the level of concordance and consistency between the two platforms, making it a suitable tool to assess the agreement in gene expression measurements across different infection conditions (see Figure 1 (objective 2)).

### 2.3 Generalized linear model and Huber regression analysis

Using Generalized Linear Models (GLMs) (Nelder and Wedderburn, 1972) and Huber regression (Maronna et al., 2019) to assess the agreement between RNA-Seq and NanoString platforms is astute. The combination offers resilience against outliers and data deviations, ensuring accurate outcomes even in the face of non-standard data assumptions. GLMs provide a flexible framework, specifically designed to evaluate the relationship between platforms such as RNA-Seq and NanoString. Each model is characterized by a Gaussian family with an identity link function.

$R^{2}$ , or the coefficient of determination, is traditionally used in Ordinary Least Squares (OLS) regression models (Dismuke and Lindrooth, 2006) to quantify how well the linear model captures the variance in the dependent variable based on the independent variables. However, when using Generalized Linear Models (GLMs), which can handle various types of distributions and do not necessarily assume a linear relationship between variables. In this case, we adapt the concept to GLMs by using what is termed Pseudo $R^{2}$ (see Eq. 1).

Huber regression (Maronna et al., 2019) was also utilized to bolster the assessment of RNA-Seq and NanoString agreement, focusing on diverse infection conditions. Given sixteen distinctive infection conditions defined by variables like virus type, treatment, and post-infection time, RNA-Seq and NanoString data were paired to form datasets. By fitting a GLM and Huber regression models to this data, we aim to discern a relationship between the two platforms that stands robust even amidst potential outliers. The metrics used for measuring Huber regression model fit are defined as follows:

$$\text{Pseudo }R^{2}=1-\frac{\sum_{i}{\left(y_{i}-{\hat{y}}_{i}\right)}^{2}}{\sum_{i}{\left(y_{i}-\text{Mean}\left(y\right)\right)}^{2}},$$ (1)

$$\text{Robust }R^{2}=1-\frac{\sum_{i}\left({\left(y_{i}-{\hat{y}}_{i}\right)}^{2}\right)}{\sum_{i}\left({\left(y_{i}-\text{Median}\left(y\right)\right)}^{2}\right)},$$ (2)

where $y_{i}$ represents the $i^{th}$ observed value, ${\hat{y}}_{i}$ represents the $i^{th}$ predicted value from the model, y is the vector containing all the observed values, Median is the median function for the Robust *R* ^2^ formula, and Mean is the mean function for the Pseudo $R^{2}$ formula.

While the Pseudo $R^{2}$ value gauges the model’s aptitude in explaining NanoString variability based on RNA-Seq inputs, the intercept and slope shed light on the fundamental relationship dynamics between the two platforms. Furthermore, the *p*-values linked to RNA-Seq coefficients are crucial. They spring from a hypothesis testing mechanism used in regression. The null hypothesis ( $H_{0}$ ) proposes no tangible relationship between RNA-Seq and NanoString for an infection condition, implying the RNA-Seq model coefficient is zero. Contrarily, the alternative hypothesis ( $H_{a}$ ) underscores a significant relationship (Huber, 1996) (see Figure 1 (objective 3)).

### 2.4 Concordance analysis

The Magnitude-Altitude Score (MAS) algorithm (Rezapour et al., 2024) offers an integral solution to identify statistically and biologically meaningful changes in gene expression across various experimental conditions. The significance of MAS lies in its integrated approach, encompassing two critical aspects of gene expression analysis: Firstly, it captures statistical significance through adjusted *p*-values, which measure the strength of evidence supporting a null hypothesis during hypothesis testing. Secondly, the MAS algorithm considers the magnitude of changes in gene expression, emphasizing its biological significance.

Definition Magnitude-Altitude Score (MAS): To find genes that have the optimal balance between ${\left|\;\right.\log_{2}(\text{FC}}_{l})|$ and $\left|{\log_{10}(p}_{l}^{BH})|\right.$ , we define *Magnitude-Altitude Score* (MAS) by

$${MAS}_{l}={\left|\;\right.\log_{2}(\text{FC}}_{l}){|}^{M}|{\log_{10}(p}_{l}^{BH}){|}^{A}$$

for l =1,2,…,s , where s is the number of rejected null hypotheses rejected based BH adjusted method, M and A are hyper-parameters (for this study, M=A=1 ).

When testing multiple hypotheses simultaneously, such as when assessing thousands of genes, there is a risk of false positives or Type I errors. The MAS algorithm addresses this challenge by employing the Benjamini–Hochberg (BH) procedure (Benjamini and Hochberg, 1995; Benjamini et al., 2009), which adjusts *p*-values to control the false discovery rate (FDR) and mitigate the issue of multiple comparisons. While statistical significance is essential, it does not provide insights into the size or direction of gene expression changes. This is where $log_{2}\left(FC\right)$ comes into play. It quantifies the effect size or the extent of a gene’s expression alteration from one condition to another, adding biological meaning to statistically significant changes.

The novelty of the MAS algorithm lies in combining these two dimensions, altitude (captured by BH-adjusted *p*-values) and magnitude (captured by $log_{2}\left(FC\right)$ ), into a unified score. This approach offers a comprehensive understanding of gene expression changes, balancing both statistical and biological significance. Genes with high MAS scores demonstrate both high statistical significance and substantial gene expression alterations, making them biologically relevant in the context of the experiment.

RNA-Seq data, which are count-based, present unique challenges due to their non-normal, often overdispersed distribution where the variance exceeds the mean (Law et al., 2014). Traditional tests like the two-sample *t*-test assume normality and equal variances, conditions not typically met by RNA-Seq data. To overcome these issues, we used Generalized Linear Models (GLMs) (Nelder and Wedderburn, 1972), which are well-suited for non-normal data types such as counts from RNA-Seq, allowing for modeling with error distributions from the exponential family, like negative binomial, ideal for addressing the discrete and overdispersed nature of the data. Within this framework, we employed the Quasi-Likelihood F-test (Wedderburn, 1974), a robust method for evaluating gene expression differences between conditions without the stringent assumptions of parametric tests. This approach directly estimates the variance-mean relationship from the data, enhancing the accuracy and reliability of inferences in complex RNA-Seq datasets and enabling the identification of differentially expressed genes by effectively handling the data’s unique distributional traits and variability.

In contrast, NanoString technology produces data that aligns with the assumptions required for the two-sample *t*-test, allowing direct application of multiple hypothesis testing using t-tests. However, for both technologies, after calculating *p*-values and log-fold changes, we applied the MAS algorithm to prioritize genes, ensuring that our analytical approach remains comprehensive and robust across different data types.

In this section, we aim to contrast the genes identified as BH-significant by the MAS algorithm in both the RNA-Seq and NanoString datasets. Initially, we apply the Generalized Linear Models with Quasi-Likelihood approach (GLMQL) to the curated RNA-Seq dataset, which includes 754 shared genes, followed by the MAS algorithm. For the NanoString data, which comprises 754 common genes, we initially conduct multiple hypothesis testing using t-tests. Subsequently, we apply the MAS algorithm to prioritize the significant findings, bypassing the use of the GLMQL framework for this dataset. Throughout the paper, the significance level ( α ) for the Benjamini–Hochberg adjusted *p*-value is set at 0.05. Our primary focus is to assess the concordance between RNA-Seq and NanoString data, with a particular emphasis on crucial infection conditions: Mock-24/72 and/or Virus-UV-24/72 (control) versus Virus-None-24/72 (experimental) (see Figure 1 (objective 4)).

Note that the MAS algorithm enables us not only to compare the number of BH-significant genes but also to assess how these genes are prioritized within each technology, enhancing our understanding of their agreement and relevance across different experimental settings.

### 2.5 Gene ontology (GO) analysis for common BH-significant transcripts across two platforms

Following the identification of common BH-significant genes across the RNA-Seq and NanoString platforms, we proceeded to conduct a Gene Ontology (GO) analysis (Young et al., 2010). This analysis was crucial for biological interpretation, especially when comparing active (Non-UV) infected samples against Mock samples at 24- and 72-h post-infection. We utilized the clusterProfiler (Wu et al., 2021) and org.Hs.eg.db (Carlson et al., 2019) packages within the R programming environment to execute the GO analysis. These tools allowed us to categorize the identified genes into groups associated with biological processes (BP), cellular components (CC), and molecular functions (MF).

The GO analysis of common BH-significant genes, which exhibit the same direction of log-fold change (LogFC) across both RNA-Seq and NanoString platforms, plays a critical role in confirming the concordance and reliability of these two distinct gene expression profiling methods. By conducting a GO analysis on this subset of genes, we focus on understanding the functional characteristics that are consistently observed regardless of the platform used. This is pivotal for several reasons:

• Validation of data consistency: The GO analysis allows us to verify that both platforms not only identify the same genes as significant under similar experimental conditions but also attribute similar biological functions to these genes. This alignment in functional attribution strengthens the validity of the results obtained from each platform and supports their use as complementary tools in gene expression analysis.
• Biological relevance: Analyzing the GO terms associated with the common significant genes helps to ensure that the significant changes in gene expression detected by both platforms are biologically meaningful. This analysis provides insight into the core biological processes and pathways that are genuinely affected by the experimental conditions, rather than being platform-specific artifacts.
• Enhanced understanding of disease mechanisms: By examining the GO processes enriched in the common significant genes, we can gain a deeper understanding of the molecular mechanisms underlying the response to viral infections.

Therefore, the GO analysis of common BH-significant genes is not just a methodological step but a fundamental part of validating the biological significance and technical consistency of the findings from our study (see Figure 1 (objective 5)).

## 3 Results

### 3.1 Correlation analysis

To assess the agreement between RNA-Seq and NanoString platforms, Spearman and Distance correlation coefficients were computed for various infection conditions. The analysis of correlation coefficients across all conditions revealed that the Spearman correlation values ranged from a minimum of 0.86 to a maximum of 0.90, while Distance correlation values varied slightly less, with a minimum of 0.86 and a maximum of 0.88. The average Spearman correlation across all conditions was approximately 0.88, indicative of a strong positive agreement. Similarly, the average Distance correlation was also around 0.87, confirming a consistent high-level agreement between the two platforms.

**FIGURE 2:**
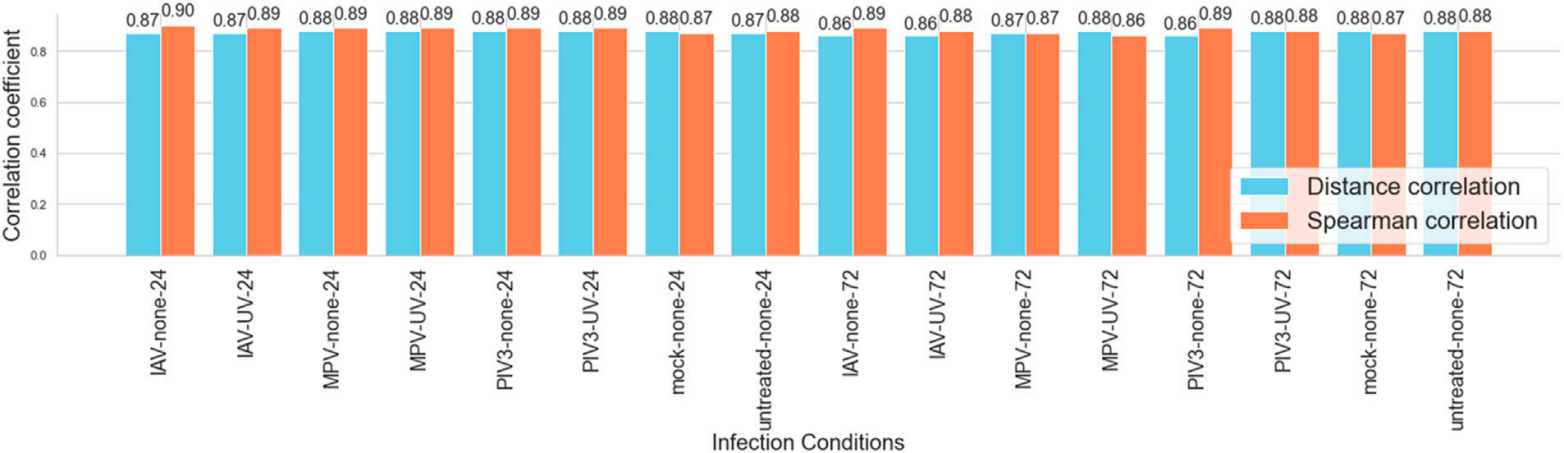
Comparison of spearman and distance correlation coefficients across infection conditions: This bar graph displays the Spearman and Distance correlation coefficients for 16 different viral infection conditions at two time points (24 and 72 h) with and without UV treatment, alongside controls. The graph emphasizes the consistently high correlation between RNA-Seq and NanoString platforms, demonstrating their robustness in capturing gene expression dynamics within the 3D airway OTE model. Correlation values range from 0.86 to 0.90 for Spearman and 0.86 to 0.88 for Distance correlation, highlighting the strong agreement across varying experimental conditions.

The maximum Spearman correlation was observed in the IAV-none-24 condition, indicating the strongest agreement under these specific experimental settings. Conversely, the minimum values for both metrics occurred in the 72-h post-infection conditions, suggesting a slight decrease in correlation as the infection progresses. Figure 2 visually represents these correlation metrics, highlighting their robust performance across different infection conditions, thereby supporting the reliability of gene expression profiling with both platforms in the study of viral infections.

### 3.2 Bland-Altman analysis

The Bland-Altman analysis was performed to evaluate the agreement between RNA-Seq and NanoString measurements of gene expression across various infection conditions. By analyzing the differences versus the averages of the two methods, we observed that the vast majority of measurements typically fell within the established limits of agreement, indicating strong concordance between the two methodologies.

In general, more than 96.6% of the measurements in each condition were within the limits, demonstrating reliable agreement across all tested scenarios. For example, in the IAV-none-24 condition, about 97.3% of the measurements were within limits, while only a small percentage were outliers. Notable genes that frequently appeared outside the limits of agreement across various conditions included TXNIP, CXCL8, and LCN2, among others. These outliers provide insight into potential areas of variability that may warrant further investigation due to biological differences or measurement discrepancies. In conditions such as IAV-none-24, MPV-none-24, and PIV3-none-24, genes like HSP90AB1, and MT2A were identified as significant outliers.

**FIGURE 3:**
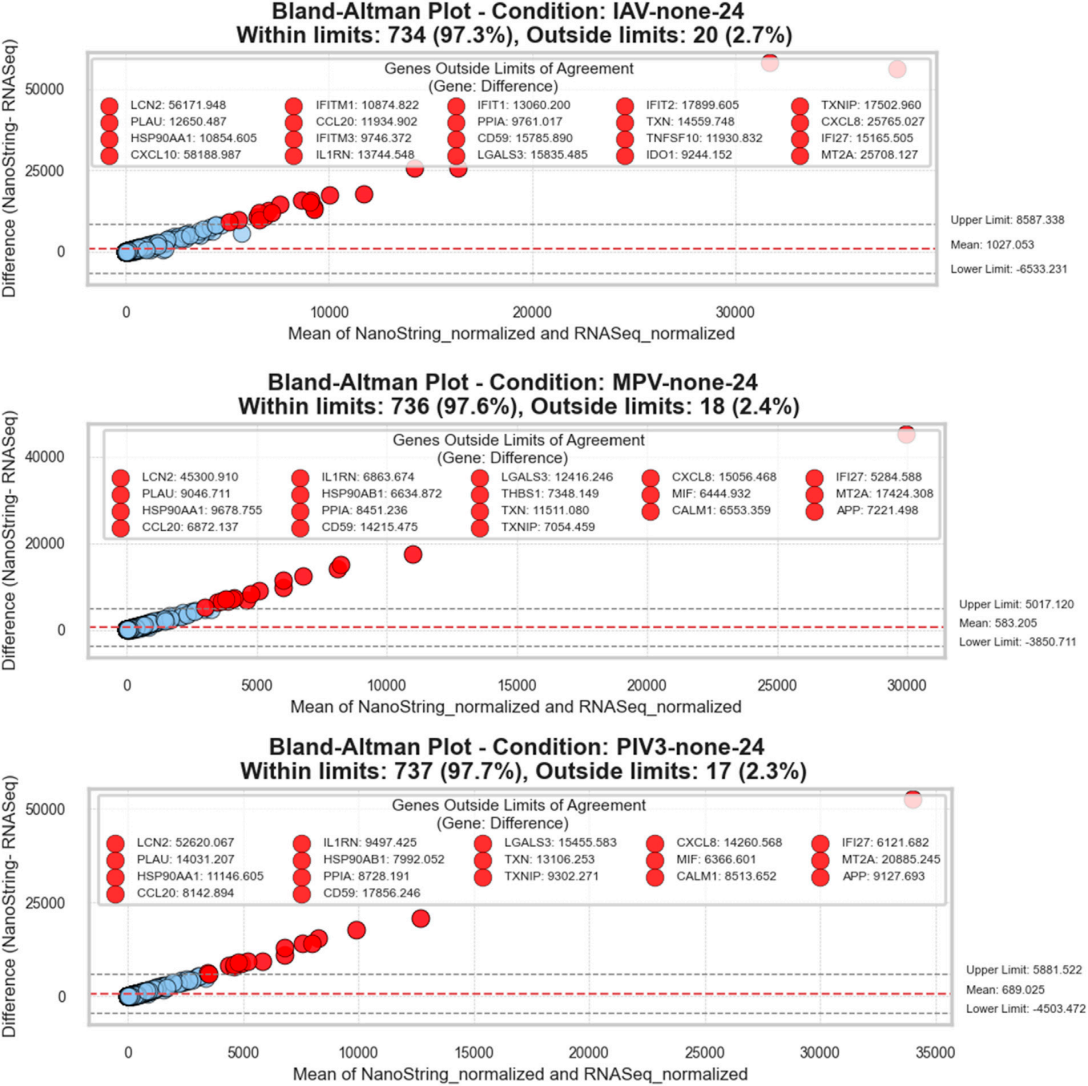
Bland-Altman plots for RNA-Seq and NanoString gene expression comparisons: This series of plots evaluates the agreement between RNA-Seq and NanoString platforms across three different conditions. Each plot visualizes the difference between the two measurement methods against their average value, facilitating a clear depiction of concordance and discrepancies. The *x*-axis represents the average of the normalized counts from both methods, while the *y*-axis displays the differences (NanoString—RNA-Seq). A dashed red line in each plot denotes the mean difference, providing an estimate of the overall bias between the methods. Dashed grey lines indicate the limits of agreement, calculated as the mean difference ± 1.96 standard deviations, representing the range within which 95% of future differences are expected to lie, assuming a normal distribution. Points are color-coded: within the limits are shown in blue, and those beyond are in red. Genes that fall outside these limits are specifically highlighted and labeled to show significant deviations. The title within each plot specifies the condition, counts the total number of genes, and notes the proportion of genes within and outside the limits of agreement, offering a succinct yet comprehensive overview of the data consistency and variation.

Figure 3 and Supplementary Figure S1 illustrate these Bland-Altman plots, providing a visual representation of the overall agreement and clearly marking the outliers. These figures help in understanding both the consistency of measurement between RNA-Seq and NanoString technologies and the specific cases where deviations occur.

### 3.3 Generalized linear model (GLM) and Huber regression analysis

We conducted Generalized Linear Models (GLM) and Huber regression to quantify the agreement between RNA-Seq and NanoString data across 16 infection conditions. These analyses provided insights into the concordance metrics such as Pseudo $R^{2}$ values, intercepts, slopes, and *p*-values.

In the GLM analysis, the Pseudo $R^{2}$ values showed a range from 0.69 in the IAV-none-72 condition to 0.89 in the Mock-24 condition, suggesting a variability in model fit that reflects the complexity of biological responses under different viral challenges. The average Pseudo $R^{2}$ value across all conditions was about 0.80, indicative of a generally good model fit. The variability was further highlighted in intercepts and slopes; intercepts varied, with the lowest being 78.54 in the IAV-UV-72 condition and the highest at 251.78 in the MPV-UV-24 condition. This variation in intercepts illustrates baseline differences in gene expression measurements between the two platforms. Slope values, which indicate the rate of change in NanoString measurements relative to RNA-Seq data, also showed substantial variation with the maximum slope observed at 8.62 in the PIV3-none-24 condition.

Huber regression reinforced these findings, with Pseudo $R^{2}$ values closely aligning with those from the GLM, affirming the robustness of the data against outliers. The consistency between the Pseudo $R^{2}$ and Robust $R^{2}$ values across the analyses emphasizes the stability of the agreement between the two platforms, even when considering the potential impact of outliers. The detailed insights into intercepts and slopes from both analyses corroborate the overall reliability and consistency of the findings, highlighting specific conditions where discrepancies were more pronounced. Tables 1, 2 compile these results comprehensively. Table 1 presents the results from the GLM analysis, including all metrics for each infection condition, while Table 2 details the outcomes from the Huber regression, highlighting how each model accommodates the outlier effects.

**TABLE 1:** Results of Generalized linear model (GLM) analysis conducted to assess the concordance between RNA-Seq and NanoString measurements across a range of infection conditions.

Condition	Pseudo $R^{2}$	Intercept	Slope	*p*-value
IAV-none-24	0.74	224.04	7.65	<0.0001
IAV-UV-24	0.85	172.46	7.97	<0.0001
MPV-none-24	0.87	158.64	7.81	<0.0001
MPV-UV-24	0.80	251.78	5.45	<0.0001
PIV3-none-24	0.86	203.44	8.62	<0.0001
PIV3-UV-24	0.86	228.40	7.77	<0.0001
Mock-24	0.89	178.85	8.02	<0.0001
Untreated-24	0.81	201.10	6.95	<0.0001
IAV-none-72	0.69	162.95	5.50	<0.0001
IAV-UV-72	0.82	78.54	6.93	<0.0001
MPV-none-72	0.82	84.24	6.15	<0.0001
MPV-UV-72	0.83	93.82	6.26	<0.0001
PIV3-none-72	0.77	130.11	5.58	<0.0001
PIV3-UV-72	0.80	91.64	5.57	<0.0001
Mock-72	0.79	119.34	7.87	<0.0001
Untreated-72	0.83	168.43	7.03	<0.0001

**TABLE 2:** Results of Huber regression analysis conducted to assess the concordance between RNA-Seq and NanoString measurements across a range of infection conditions.

Condition	$\text{Pseudo }R^{2}$	$\text{Robust }R^{2}$	Intercept	Slope	*p*-value
IAV-none-24	0.73	0.80	49.80	6.93	<0.0001
IAV-UV-24	0.84	0.71	32.54	7.60	<0.0001
MPV-none-24	0.86	0.73	41.58	7.20	<0.0001
MPV-UV-24	0.73	0.75	38.53	7.07	<0.0001
PIV3-none-24	0.86	0.80	36.05	8.88	<0.0001
PIV3-UV-24	0.85	0.78	38.25	8.09	<0.0001
Mock-24	0.89	0.76	46.80	7.71	<0.0001
Untreated-24	0.79	0.79	41.08	7.94	<0.0001
IAV-none-72	0.69	0.78	35.49	6.00	<0.0001
IAV-UV-72	0.80	0.80	27.03	6.12	<0.0001
MPV-none-72	0.82	0.78	28.90	6.07	<0.0001
MPV-UV-72	0.82	0.70	30.78	6.01	<0.0001
PIV3-none-72	0.76	0.75	29.70	5.81	<0.0001
PIV3-UV-72	0.80	0.74	21.37	5.52	<0.0001
Mock-72	0.79	0.73	38.69	7.50	<0.0001
Untreated-72	0.83	0.75	45.11	6.86	<0.0001

### 3.4 Concordance analysis

The concordance analysis revealed varied results across different experimental conditions, highlighting the unique response profiles under various viral challenges and controls. For example, the comparison between the IAV-UV-24 (as baseline) and IAV-none-24 conditions identified a significant number of genes by both RNA-Seq (357 genes) and NanoString (333 genes), with 169 genes common to both datasets. However, in contrasts such as MPV-none-24 against MPV-UV-24 and Mock-24, the RNA-Seq analysis did not identify any significant genes, resulting in no common significant genes between the RNA-Seq and NanoString platforms. This suggests either a minimal gene expression response to MPV or limitations in the sensitivity of RNA-Seq under these experimental settings.

**FIGURE 4:**
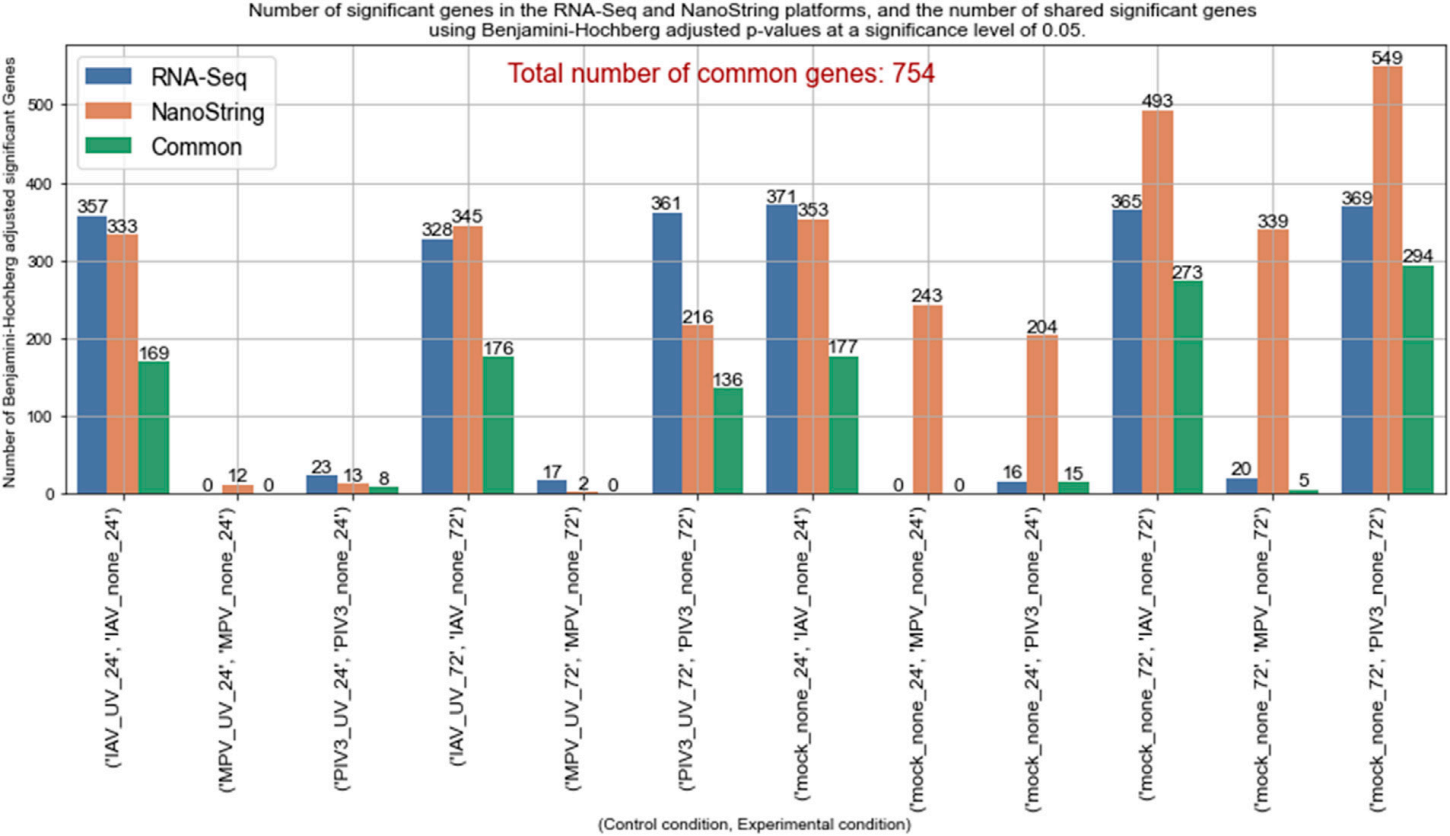
Comparison of MAS significant genes between RNA-Seq and NanoString platforms across various infection conditions.

Contrasts like IAV-none-72 against Mock-72 showed a substantial overlap with 273 common genes, reflecting a strong and consistent gene expression response to IAV across different controls at 72 h, which was the highest number of common significant genes observed among all comparisons. The contrast PIV3-none-72 against PIV3-UV-72 demonstrated that the RNA-Seq identified 361 significant genes, while NanoString identified 216, with 136 genes common between them. This notable difference highlights the specific sensitivity of each platform under varying experimental conditions. Figure 4 presents a comparison of significant genes identified using the MAS algorithm in both RNA-Seq and NanoString datasets across various infection conditions.

**FIGURE 5:**
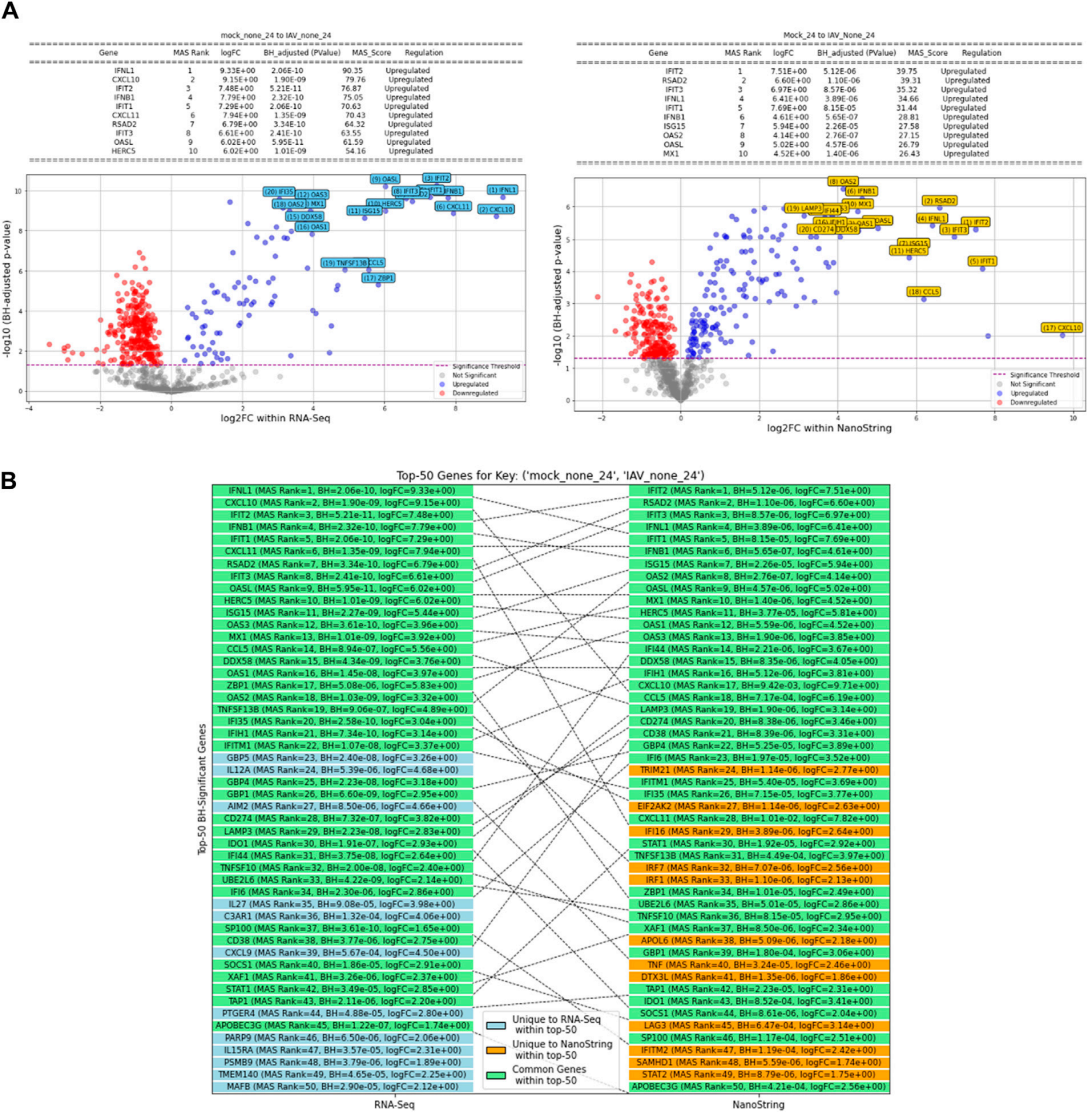
**(A)** Volcano plot comparing gene expression between Mock-24 and IAV-None-24 in RNA-seq and NanoString datasets (Control: Mock-24, Experimental: IAV-None-24). **(B)** Top 50 differentially expressed genes identified using the MAS algorithm, emphasizing the overlapping genes between RNA-Seq and NanoString platforms (Control: Mock-24, Experimental: IAV-None-24).

Volcano plots illustrating gene expression contrasts between multiple control and experimental groups within the RNA-Seq and NanoString datasets are provided in Supplementary Figure S2, with a specific case showcased in Figure 5A. Moreover, Supplementary Figure S3 displays the top 50 differentially expressed genes identified using the MAS algorithm, highlighting the common genes between the RNA-Seq and NanoString platforms within this top-50 list. A specific example of this is showcased in Figure 5B. This figure presents the top 50 differentially expressed genes, emphasizing the overlapping genes identified by both RNA-Seq and NanoString platforms when comparing the control condition (Mock-24) to the experimental condition (IAV-None-24). The diagram categorizes genes that are unique to each platform and those that are common between them, providing a clear visual representation of the concordance in gene expression detection across these two technologies. This overlap underscores the reliability of the identified significant genes and highlights the strengths of integrating data from multiple platforms to achieve a comprehensive understanding of gene expression changes.

### 3.5 Gene ontology (GO) analysis for common BH-significant transcripts across two platforms

**FIGURE 6:**
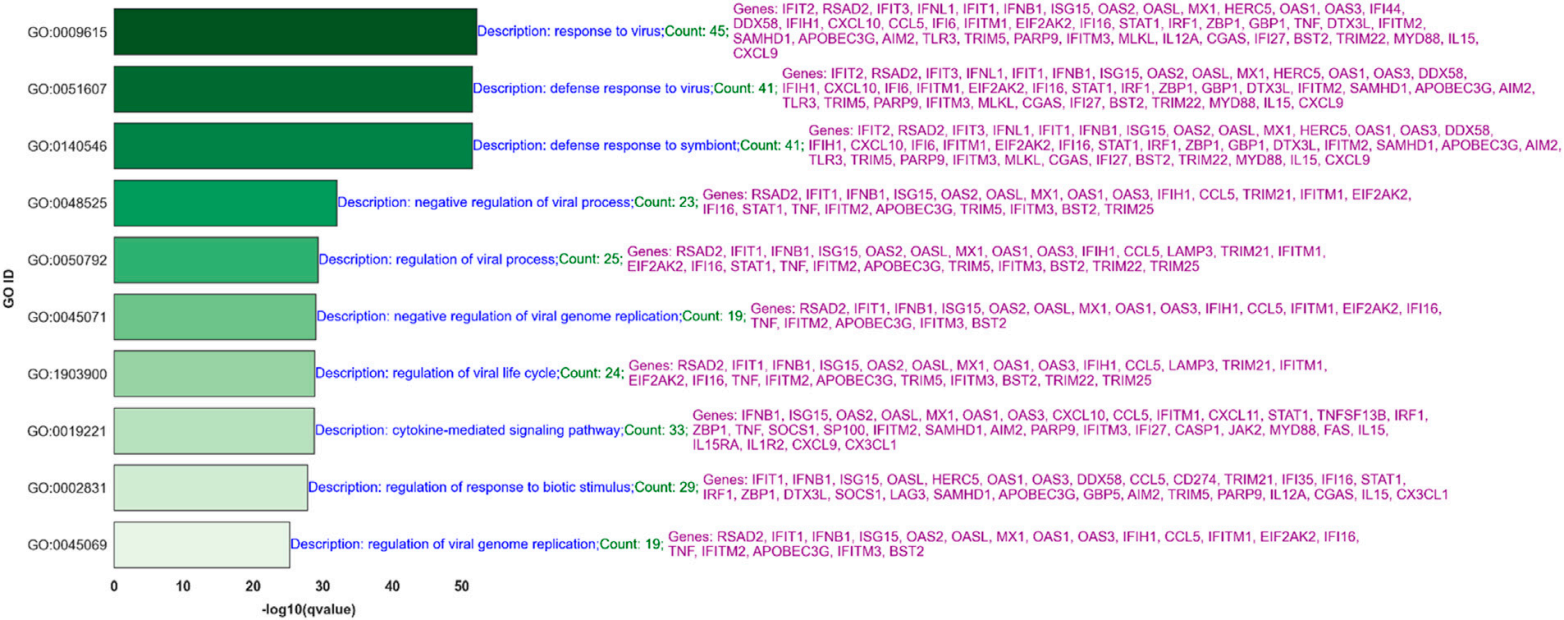
Top 10 Significant Gene Ontology (GO) Processes for Upregulated Genes in IAV-None-24 versus Mock-24 Comparison: This bar graph ranks the top 10 out of 567 significant GO processes based on q-values for BH-significantly upregulated genes identified in the IAV-None-24 versus Mock-24 comparison across both RNA-Seq and NanoString platforms. Each bar represents a GO process, with its length corresponding to the -log10 (q-value), indicating the statistical significance. The processes include “response to virus,” “defense response to virus,” and “type I interferon signaling pathway,” among others. The descriptions highlight the number of genes involved and key genes associated with each process, such as IFIT2, RSAD2, IFIT3, IFNL1, and ISG15, demonstrating the robust antiviral and immune response elicited by IAV infection.

Figure 6 presents the top 10 out of 567 significant Gene Ontology (GO) processes based on q-values for BH-significantly upregulated genes in the IAV-None-24 versus Mock-24 comparison across both platforms. Key processes related to IAV infection include the response to the virus, defense response to the virus, type I interferon signaling pathway, regulation of cytokine-mediated signaling pathway, and cellular response to interferon-gamma. This analysis aims to answer how the gene expression changes observed align with known biological processes and whether these changes are statistically and biologically significant. For example, the process “response to virus” includes 45 genes such as IFIT2, RSAD2, and IFIT3, indicating a robust antiviral response. Similarly, the “defense response to virus” process, which shares many genes with the “response to virus” process, highlights the immune system’s multifaceted approach to combating viral infections.

**FIGURE 7:**
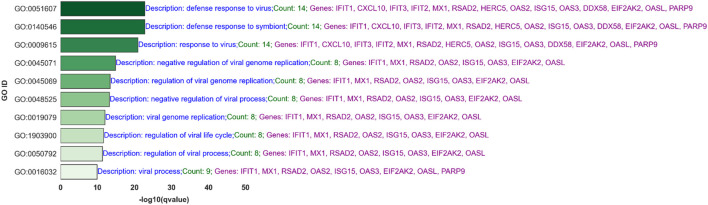
Top 10 Significant Gene Ontology (GO) Processes for Upregulated Genes in PIV3-None-24 versus Mock-24 Comparison: This bar graph ranks the top 10 out of 92 significant GO processes based on q-values for BH-significantly upregulated genes identified in the PIV3-None-24 versus Mock-24 comparison across both RNA-Seq and NanoString platforms.

Processes like the “negative regulation of viral process” and “regulation of viral process,” which include genes such as RSAD2, IFIT1, and ISG15, suggest mechanisms by which the host cells attempt to limit viral replication and spread. Moreover, pathways like “type I interferon signaling” and “response to interferon-gamma” underscore the critical role of interferons in the antiviral defense, involving key signaling molecules such as STAT1 and IFIH1. Figure 7 shows the top 10 of 92 significant GO processes for BH-significantly upregulated genes in the PIV3-None-24 versus Mock-24 comparison across both platforms, highlighting processes relevant to PIV3 infection such as defense response to virus, regulation of viral genome replication, and positive regulation of interferon-beta production (Supplementary Table S4 provides the top 20 significant GO processes).

**FIGURE 8:**
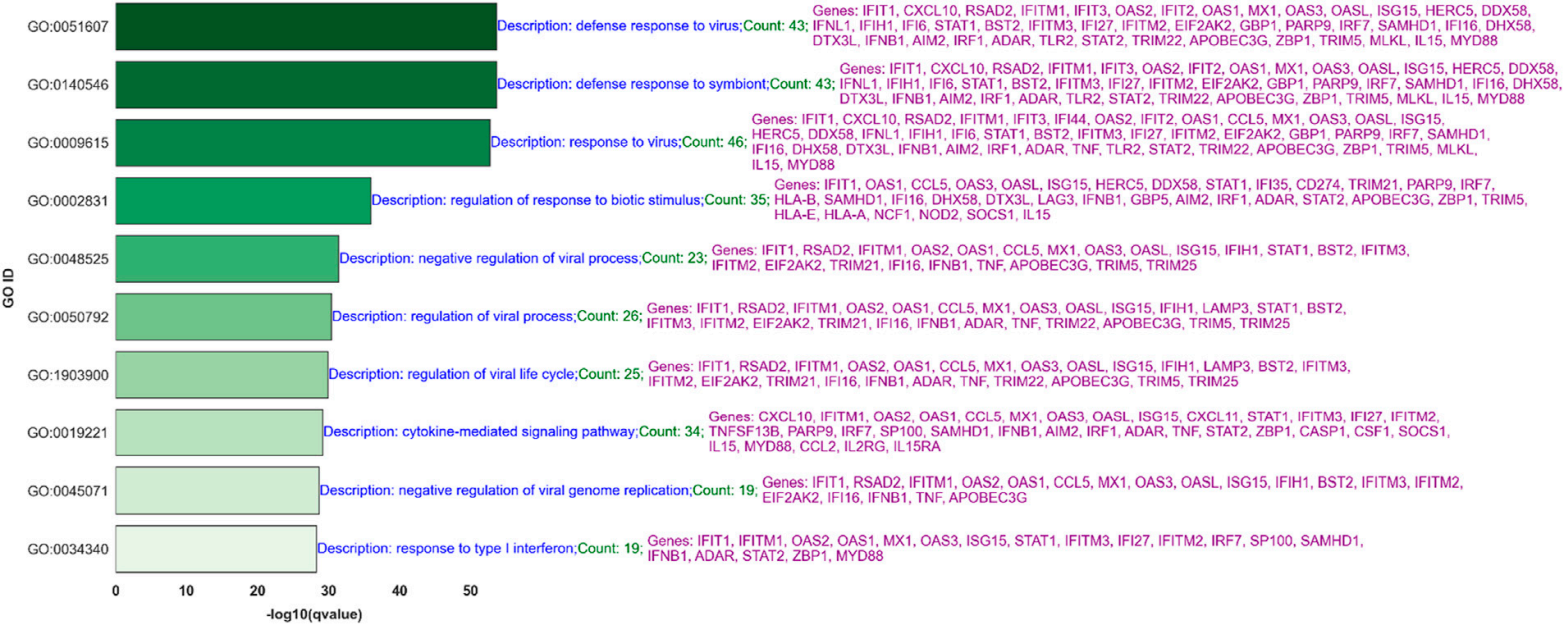
Top 10 Significant Gene Ontology (GO) Processes for Upregulated Genes in IAV-None-72 versus Mock-72 Comparison: This bar graph ranks the top 10 out of 573 significant GO processes based on q-values for BH-significantly upregulated genes identified in the IAV-None-72 versus Mock-72 comparison across both RNA-Seq and NanoString platforms. Each bar represents a GO process, with its length corresponding to the -log10 (q-value), indicating the statistical significance. The descriptions highlight the number of genes involved and key genes associated with each process, such as IFIT1, CXCL10, and RSAD2, demonstrating the host’s robust antiviral and immune response.

**FIGURE 9:**
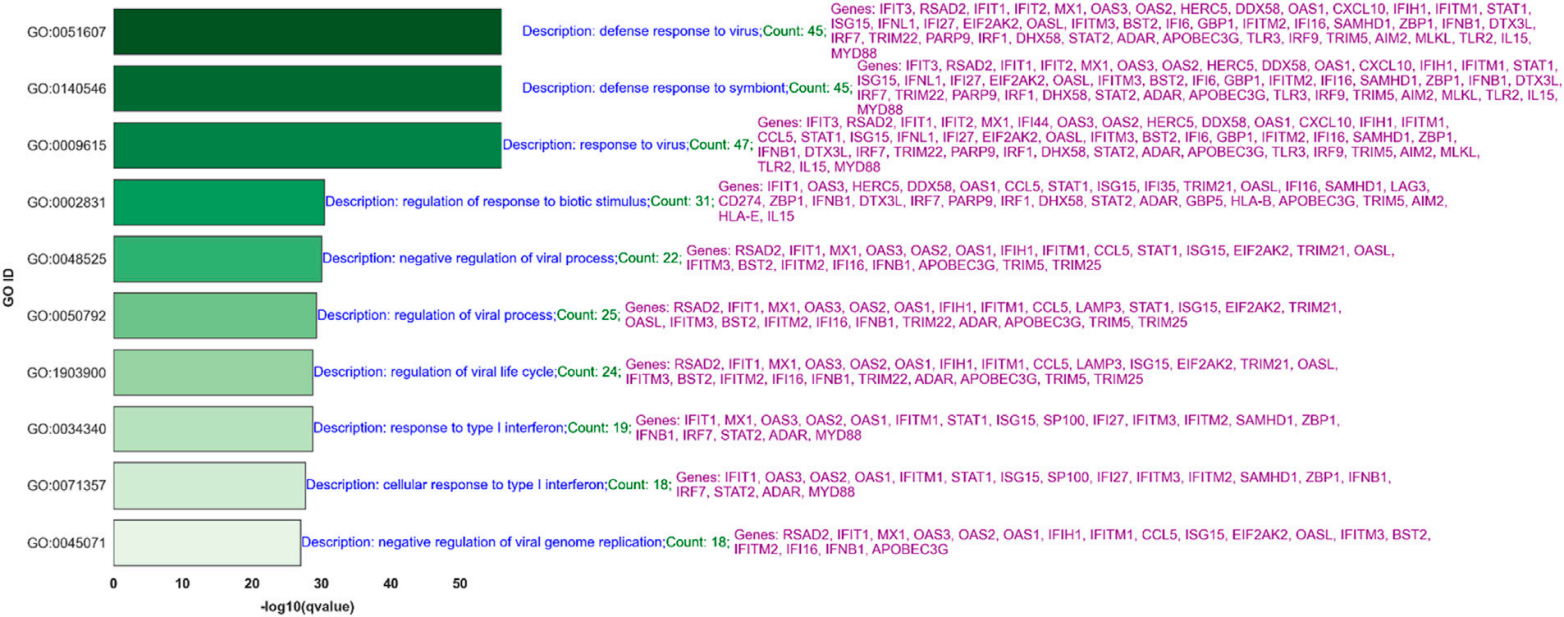
Top 10 Significant Gene Ontology (GO) Processes for Upregulated Genes in PIV3-None-72 versus Mock-72 Comparison. This bar graph ranks the top 10 out of 464 significant GO processes based on q-values for BH-significantly upregulated genes identified in the PIV3-None-72 versus Mock-72 comparison across both RNA-Seq and NanoString platforms.

Figure 8 displays the top 10 of 573 significant GO processes, based on q-values for BH-significantly upregulated genes in the IAV-None-72 versus Mock-72 comparison across both platforms (Supplementary Table S5 provides the top 20 significant GO processes). Supplementary Table S6 provides the top 20 significant GO processes, based on q-values for BH-significantly upregulated genes in the MPV-None-72 versus Mock-72 comparison across both platforms. Figure 9 illustrates the top 10 of 464 significant GO processes for BH-significantly upregulated genes in the PIV3-None-72 versus Mock-72 comparison across both platforms (Supplementary Table S7 provides the top 20 significant GO processes).

The comparison of GO processes across Figures 6–9, based on common significant genes identified by both RNA-Seq and NanoString platforms, provides a nuanced understanding of the host’s immune response to various viral infections. In Figure 6, for IAV-None-24 versus Mock-24, the predominant processes include “response to virus” and “defense response to virus,” highlighting a robust early immune reaction. This pattern is consistent in Figure 8 for the IAV-None-72 versus Mock-72 comparison, suggesting a sustained antiviral response over a longer period. Conversely, Figure 7, which compares PIV3-None-24 to Mock-24, shows a similar but less pronounced response, with fewer genes involved in the “defense response to virus” process, indicating a less intense early response compared to IAV. In Figure 9, the PIV3-None-72 versus Mock-72 comparison reveals an increased involvement of genes in the “defense response to virus” process, such as IFIT1 and OAS3, indicating an amplified immune response as the infection progresses.

## 4 Discussion

### 4.1 Correlation analysis

The results of the correlation analysis underscore the strong agreement between RNA-Seq and NanoString technologies in capturing gene expression profiles within a 3D airway OTE model under various viral infection conditions. Both platforms exhibited high correlation coefficients, with Spearman and Distance correlations consistently ranging from 0.86 to 0.90 across different experimental setups and time points (see Figure 2). This robust consistency validates the use of these technologies as reliable tools for detailed and accurate gene expression analysis, suitable for both broad and targeted studies.

The consistency observed in both UV-treated and non-UV-treated samples further highlights the platforms’ capacity to accurately reflect the impact of viral infections on cellular machinery, independent of viral replication. This insight is critical for understanding the mechanisms of viral entry and the subsequent activation of innate immune responses, as seen with the upregulation of specific interferon-stimulated genes.

Overall, the correlation analysis study not only demonstrates the technical reliability and complementarity of RNA-Seq and NanoString in a 3D model but also reinforces the utility of these platforms in advancing our understanding of viral pathogenesis and host response. This contributes significantly to the field of virology and respiratory biology, providing a robust foundation for future research into therapeutic interventions and the biological underpinnings of disease.

#### 4.1.1 Findings based on correlation analysis

The correlation analysis study conclusively demonstrates that both RNA-Seq and NanoString are robust and reliable platforms for gene expression analysis, showing strong correlation across various conditions in a 3D airway OTE model. These findings affirm the applicability of these technologies in capturing detailed and accurate gene expressions during viral infections, making them valuable tools for virology research and beyond. This consistency, evident in both UV and non-UV treated samples across critical time points, highlights their potential in a wide array of biomedical applications, enhancing our understanding of complex biological responses to viral challenges.

### 4.2 Bland-Altman analysis

Bland-Altman plots showed (see Figure 3; Supplementary Figure S1) that more than 96.6% of genes had differences within acceptable limits between NanoString and RNA-Seq data, indicating good agreement between the two techniques. However, a positive linear trend was observed, meaning that the difference between the two measurements increased with the average of the measurements, and NanoString measurements were higher than RNA-Seq for genes with higher expression levels.

This trend is due to different normalization techniques used for each dataset. RNA-Seq data were normalized using the TMM method, which scales library sizes based on the assumption that most genes are not differentially expressed. This method effectively handles RNA composition bias. In contrast, NanoString data were normalized through a two-step process: a positive control normalization factor that adjusts for variations across samples, lanes, cartridges, and days due to various factors, and a CodeSet content normalization factor using reference genes to adjust for differences in analyte abundance or quality.

Despite the observed trend, the level of agreement between the two platforms was high, with a maximum of 3.4% of genes falling outside the limits of agreement across all conditions. Both RNA-Seq and NanoString technologies provide broadly consistent gene expression measurements, despite their differences in detection and normalization techniques.

#### 4.2.1 Findings based on Bland-Altman Analysis

He Bland-Altman analysis conducted across various infection conditions and time points substantiates a high level of agreement between RNA-Seq and NanoString platforms, with the vast majority of gene expression measurements falling within the agreed limits of concordance. Specifically, over 96.6% of genes for the paired measurements in each condition are within the limits, indicating minimal bias and confirming the reliability of these methods for comparative gene expression studies. The small percentage of measurements outside the limits suggests only minor discrepancies, likely due to the inherent differences in the technologies. This high degree of concordance underpins the robustness of the 3D airway OTE model as a reliable system for studying complex biological interactions, such as those involved in viral infections, using either of these gene expression platforms.

### 4.3 Generalized linear model and huber regression analysis

Across all infection conditions, both the GLM and Huber regression analyses consistently indicated high Pseudo $R^{2}$ values, ranging between 0.69 and 0.89 (see Tables 1, 2). The $\text{Pseudo }R^{2}$ values signify the proportion of variability in NanoString measurements that can be explained by RNA-Seq data. A Pseudo $R^{2}$ value above 0.70 generally indicates a strong linear relationship. Therefore, it is evident that the RNA-Seq readings significantly explain the variation observed in the NanoString measurements across the variety of infection conditions tested.

In the Huber Regression, the Robust $R^{2}$ values, which provide insight in the presence of outliers by focusing on median deviations, predominantly range between 0.70 and 0.80. This suggests that the Huber model consistently captures the central tendencies of the data across various conditions. However, the disparity between Robust $R^{2}$ and Pseudo $R^{2}$ in certain conditions underscores the subtle differences in data distribution and the potential presence of outliers.

For all conditions, the *p*-values are less than 0.0001. This reaffirms the statistical significance of the relationship between RNA-Seq and NanoString across all infection conditions. This small *p*-value contradicts the null hypothesis that posits no tangible relationship between RNA-Seq and NanoString measurements. Instead, the data strongly supports the alternative hypothesis, asserting a significant relationship between the two platforms across different infection conditions. The intercepts and slopes between the two methods vary, but not drastically, which might be attributed to the robustness offered by the Huber regression against outliers.

#### 4.3.1 Findings based on regression analysis

The comprehensive analysis utilizing GLMs and Huber regression has effectively demonstrated a high degree of agreement between RNA-Seq and NanoString platforms across various infection conditions. Both analytical approaches reveal significant consistency, with high Pseudo $R^{2}$ values indicating strong model fits and minimal variation unexplained by the models. The remarkably low *p*-values across all conditions confirm the statistical significance of the relationship between the datasets. These results highlight the robustness of both RNA-Seq and NanoString in capturing and reflecting gene expression dynamics in a biologically relevant model, underscoring their reliability and interchangeability in genomic studies focused on viral infections. The use of GLMs and Huber regression not only enhances confidence in these findings by addressing potential outliers and non-standard data distributions but also solidifies the foundational analytical framework for future comparative studies in molecular biology.

### 4.4 Concordance analysis

The efficacy of the MAS algorithm is prominently reflected in the comparative analysis between RNA-Seq and NanoString datasets across various infection conditions (see Figures 4, 5, as well as Supplementary Figures S3, S4). The most prominent outcome observed was for the (IAV-UV-24, IAV-none-24) and (IAV-UV-72, IAV-none-72) conditions with IAV-UV-24 and IAV-UV-72 serving as baselines, and IAV-none-24 and IAV-none-72 as the corresponding experimental conditions. In these instances, both datasets presented a substantial number of significant genes, with a significant overlap in the findings. This suggests a reliable detection and agreement between both methods, emphasizing the robustness of the MAS algorithm. Contrastingly, for the condition (MPV-UV-24, MPV-none-24), while the RNA-Seq dataset showed no significant genes, the NanoString dataset detected 12. The absence of common genes further accentuates the distinct sensitivities or potential discrepancies between the two platforms. This may warrant further investigation into the nature of these discrepancies (see Figure 4).

Interestingly, under conditions (Mock-24, MPV-None-24) and (Mock-24, PIV3-None-24), the NanoString dataset identified a notably higher number of genes compared to RNA-Seq. This may suggest a heightened sensitivity of the NanoString platform or potential false discoveries, emphasizing the necessity of integrated analysis using MAS. A similar trend was observed under the conditions (Mock-72, IAV-None-72), (Mock-72, MPV-None-72), and (Mock-72, PIV3-none-72). The NanoString consistently identified more genes, yet there was a significant overlap in findings, particularly for the IAV and PIV3 conditions. This suggests that while both platforms have unique sensitivities, there is still substantial concordance in their findings when analyzing complex infection conditions (see Figure 4).

Supplementary Figures S3, S4 and Figure 5 underscore the strong alignment between the RNA-Seq and NanoString platforms in identifying significant genes. This congruence is evident across most instances, with the exception of the MPV virus. The set of genes discerned by both platforms offers an extensive overview of immune regulation and cellular defense processes. At the heart of the antiviral response lie genes such as ISG15, MX1, RSAD2, and the OAS family members (OAS1, OAS2, OAS3, and OASL). These genes are instrumental in recognizing viral infiltration, marshaling antiviral defenses, and orchestrating RNA degradation in affected cells. Concurrently, DDX58 and IFIH1 emerge as sentinel detectors of viral RNA, activating cellular defense mechanisms. Meanwhile, the IFIT proteins (IFIT1, IFIT2, IFIT3), as products of interferon stimulation, are recognized for curtailing viral replication. CXCL10 and CXCL11 highlight the proactive immune response by drawing immune cells to infection or inflammation sites.

#### 4.4.1 Findings based on concordance analysis

The Concordance Analysis using the Magnitude-Altitude Score (MAS) algorithm effectively demonstrated the agreement and discrepancies between RNA-Seq and NanoString datasets across a variety of infection conditions. Notably, significant gene overlap in conditions like (IAV-UV-24, IAV-none-24) and (IAV-UV-72, IAV-none-72) confirmed the MAS algorithm’s reliability in identifying biologically relevant changes. However, distinct outcomes such as in (Mock-24, MPV-none-24), where RNA-Seq identified no significant genes while NanoString detected several, underscore the differences in sensitivity between the two platforms. This variation, along with NanoString’s consistently higher gene detection in other conditions, points to its increased sensitivity or potential for false positives, highlighting the importance of using MAS to integrate findings comprehensively. These results not only affirm the strengths and limitations of each platform but also reinforce the value of a combined analytical approach to ensure thorough and meaningful insights into gene expression changes in the context of infectious diseases.

### 4.5 Gene ontology (GO) analysis for common BH-significant transcripts across two platforms

#### 4.5.1 GO processes for IAV at 24 h post-infection

In analyzing the top 30 (out of 567) significant Gene Ontology (GO) processes at 24 h post-infection with IAV (see Figure 6 for the top 10 GO processes), we observed several crucial biological responses. These findings underscore the robustness of our experimental design and the concordance between RNA-Seq and NanoString platforms. Predominantly, processes such as “response to virus” and “defense response to virus” were notably upregulated, involving key antiviral genes like IFIT2, RSAD2, and MX1, among others. These genes play critical roles in the antiviral response, illustrating a broad and dynamic regulatory network activated upon viral infection.

Furthermore, processes like “negative regulation of viral process” and “regulation of viral genome replication” were also prominent, featuring genes such as OAS1 and EIF2AK2, which are essential for inhibiting viral replication and modulating the immune response. This not only confirms the biological impact of IAV infection on cellular machinery but also highlights the effectiveness of our normalization and analytical approaches in capturing these subtle yet significant changes.

The presence of genes across multiple related GO categories, such as IFNB1 and STAT1 in both the “type I interferon signaling pathway” and “response to interferon-gamma,” validates the consistency across data platforms and underscores the interconnected nature of immune responses. These findings are crucial for understanding the mechanism of action of IAV and potentially guiding therapeutic interventions.

The involvement of these specific pathways and genes across different categories supports the reliability of our data and underscores the biological relevance of our findings. Such consistency in data across different technological platforms not only strengthens the validity of the results but also demonstrates the robust nature of the experimental design and analysis, thus enhancing our confidence in these platforms’ ability to accurately reflect biological realities under infection conditions.

#### 4.5.2 GO processes for PIV3 at 24 h post-infection

The analysis of the top 30 (out of 92) significant GO processes for PIV3 at 24 h post-infection (see Figure 7 for the top 10 GO processes) reveals a robust activation of antiviral immune responses, underlining the concordance between the RNA-Seq and NanoString platforms. Notably, processes like “defense response to virus” and “response to virus” prominently feature genes such as IFIT1, MX1, and RSAD2, which are critical in mediating cellular defenses against viral replication and propagation.

Further analysis shows significant upregulation in “negative regulation of viral genome replication” and related processes like “regulation of viral genome replication” and “viral genome replication” itself, indicating a strong cellular attempt to control and mitigate viral replication. This is underscored by the involvement of OAS2 and EIF2AK2, which are known to play key roles in the viral defense mechanism by degrading viral RNA and inhibiting viral protein synthesis.

Moreover, pathways such as “positive regulation of interferon-beta production” and the broader “type I interferon production” pathway highlight the cellular response to PIV3 infection. The presence of genes like OAS2 and DDX58 in these pathways reflects the cell’s efforts to activate and propagate antiviral signaling cascades that enhance the immune response.

This comprehensive engagement of antiviral response genes across multiple GO categories not only confirms the biological impact of PIV3 infection on cellular functions but also demonstrates the technical consistency and reliability of our experimental platforms. By capturing these gene expression changes across both RNA-Seq and NanoString technologies, our findings reinforce the validity of the data and the biological insights they provide.

Such detailed mapping of antiviral responses at the molecular level is crucial for understanding the dynamics of PIV3 infection and potentially guiding the development of targeted therapeutic strategies. The alignment of significant gene responses across different technological platforms further supports the robustness of our study and the biological relevance of the observed changes in gene expression.

#### 4.5.3 GO processes for IAV at 72 h post-infection

The analysis of the top 30 (out of 573) significant Gene Ontology (GO) processes at 72 h post-infection with IAV (see Figure 8 for the top 10 GO processes) provides a comprehensive view of the ongoing immune responses and adaptations to prolonged viral exposure. Key processes such as “defense response to virus” and “response to virus” prominently feature an array of genes including IFIT1, MX1, and STAT1, which are integral to antiviral defenses, showcasing a sustained immune activation over time.

Significant upregulation in processes like “negative regulation of viral process” and “regulation of viral genome replication” highlights the ongoing cellular efforts to control and mitigate viral replication. This includes the action of genes such as OAS1 and EIF2AK2, crucial for breaking down viral RNA and inhibiting the viral replication machinery, demonstrating the cells’ adaptive responses to continued viral presence.

Additionally, the “cytokine-mediated signaling pathway” remains a critical component of the immune response, with genes such as CXCL10 and IFNB1 playing roles in signaling cascades that regulate inflammation and immune cell recruitment. The persistence of this pathway at 72 h illustrates the body’s continued effort to mobilize and coordinate immune defenses against IAV.

Moreover, the involvement of the “type I interferon signaling pathway” with genes like IRF7 and STAT2 highlights the role of interferon responses in modulating the immune landscape during the later stages of infection. The sustained expression of these genes indicates a robust antiviral state that extends well beyond the initial infection phase.

This rich data not only underscores the complexity of the immune response to IAV but also demonstrates the depth of biological insights gleaned from high-throughput gene expression platforms. The overlap in significant genes across both RNA-Seq and NanoString platforms lends high confidence to these observations, affirming the reliability of our methodologies in capturing biologically relevant changes. These insights are crucial for understanding the dynamics of IAV infection over time and could be pivotal in designing strategies for intervention and therapy.

#### 4.5.4 GO processes for PIV3 at 72 h post-infection

The examination of the top 30 (out of 464) significant Gene Ontology (GO) processes at 72 h post-infection with Parainfluenza virus 3 (PIV3) (see Figure 9 for the top 10 GO processes) illustrates a persistent and complex immune response. Key processes such as “defense response to virus” and “response to virus” continue to feature prominently, with a broad array of immune response genes including IFIT1, MX1, and STAT1. These genes are crucial for continuing the antiviral response, highlighting the body’s ongoing efforts to combat viral persistence.

Significantly, processes like “negative regulation of viral process” and “regulation of viral genome replication” indicate an active cellular mechanism to suppress viral replication. This involves genes such as RSAD2 and EIF2AK2, which are vital for inhibiting viral proliferation and mitigating viral impacts at cellular levels.

Moreover, the “type I interferon signaling pathway” and “response to type I interferon” are notably active, involving genes like IRF7 and STAT2. These pathways play a critical role in orchestrating a broad antiviral state, which is essential for controlling and possibly resolving the viral infection. The sustained activation of these pathways suggests a robust antiviral signaling that adapts over the course of the infection.

Additionally, the “cytokine-mediated signaling pathway” underscores the ongoing communication between immune cells, with cytokines like CXCL10 and IFNB1 playing pivotal roles in modulating the immune landscape. This continued cytokine signaling is crucial for maintaining an effective immune response and potentially initiating recovery processes.

The persistence of these immune processes over 72 h demonstrates not only the dynamic nature of the host response to PIV3 but also validates the reliability of our data across RNA-Seq and NanoString platforms. This consistency provides confidence in the biological relevance of the observed gene expressions and supports the robustness of the experimental and analytical methodologies used. Understanding these dynamics is essential for developing therapeutic strategies that can effectively target the later stages of viral infections, providing insights into potential interventions to modulate or enhance the immune response.

#### 4.5.5 Changes in GO processes over time

##### 4.5.5.1 Changes in GO processes over time for IAV

From the 24-h to the 72-h time points, we observe a notable expansion in the number of genes involved in significant GO processes, such as “defense response to virus,” which increases from 41 to 43 genes, and “response to virus,” which grows from 45 to 46 genes. This suggests a broadening and intensification of the immune response as the infection progresses.

Key processes like “negative regulation of viral process” and “regulation of viral genome replication” maintain their importance over time, demonstrating the body’s ongoing efforts to control viral replication and activity. The “cytokine-mediated signaling pathway” expands from 33 to 34 genes, highlighting the increased communication within the immune system to coordinate a comprehensive response to the viral infection.

The “response to type I interferon” and “type I interferon signaling pathway” show increased gene involvement, indicating a sustained focus on interferon-driven defense mechanisms crucial for antiviral defense. By 72 h, there’s a marked engagement in “response to interferon-gamma” and “response to interferon-beta,” pointing to a robust activation of various interferon responses essential for modulating the broader immune reaction.

The transition from immediate, targeted antiviral actions to a broader, more systemic immune response is characterized by the increased involvement of genes in cytokine signaling and interferon responses. This evolution suggests that while the immediate response aims to contain the virus, the prolonged response prepares the body for sustained defense, potentially against ongoing or secondary viral attacks. The significant involvement of Interferon-Stimulated Genes (ISGs) like ISG15, OAS1, and MX1 throughout the infection period underscores the critical role of interferon responses. Understanding these dynamic changes in gene expression and immune pathway activation can guide the development of antiviral therapies, enhancing their effectiveness by timing administration to coincide with peak expressions of key immune responses.

##### 4.5.5.2 Changes in GO processes over time for MPV

For MPV, RNA-Seq technology did not reveal any BH significant genes at 24 h post-infection, despite the utilization of stringent multiple hypothesis testing that was confined to common genes rather than the entire gene dataset. This outcome highlights a possible limitation of RNA-Seq in detecting early gene expressions in response to MPV infection, potentially due to the low abundance or subtle expression changes of these genes that fall below the detection sensitivity of RNA-Seq. By 72 h post-infection, although only five common genes (IFI44, OASL, OAS3, IRF9, IRF7) are identified, these genes contribute significantly to a range of crucial GO processes, emphasizing the depth of the immune response despite the limited number of genes involved. The top processes identified include:

• Response to virus and defense mechanisms: All five genes are involved in the “response to virus” process, with four contributing to “defense response to virus.” This shows a targeted activation of antiviral defense mechanisms.
• Pattern recognition receptor signaling pathways: Multiple processes such as “cytoplasmic pattern recognition receptor signaling pathway in response to virus” and “pattern recognition receptor signaling pathway” are notably activated, indicating a sophisticated cellular recognition of viral components.
• Regulation of interferon and viral replication: Significant involvement in “regulation of type I interferon-mediated signaling pathway” and “negative regulation of viral genome replication” demonstrates the cellular strategies to enhance antiviral defenses and inhibit viral propagation.

Note that when the BH adjustment was applied across the entire set of 19,671 genes tested by RNA-Seq for both 24- and 72-h post-infection, all genes failed to pass the BH significance threshold. This outcome indicates that when considering the broader and more variable context of the full gene set, the individual *p*-values did not achieve statistical significance after adjusting for the large number of comparisons.

The case of MPV illustrates the potential superiority of NanoString over RNA-Seq for certain viral infections where early and accurate detection of a small number of genes is critical. NanoString’s methodology allows for a more direct and quantifiable approach, making it exceptionally useful in cases where the virus may evade early detection or suppress initial immune responses, as observed with MPV. This technology enhances the ability to detect subtle yet significant changes in gene expression that are pivotal in the early stages of viral infection.

##### 4.5.5.3 Changes in GO processes over time for PIV3

Between the 24-h and 72-h time points, there is a notable increase in the number of genes involved in significant GO processes. For instance, “defense response to virus” initially involves 14 genes at 24 h, which significantly expands to 45 genes by 72 h. This suggests a more robust and diversified immune response as the infection progresses.

At 24 h, processes like “negative regulation of viral genome replication” and “regulation of viral genome replication” are primarily focused on immediate viral suppression, involving 8 genes. By 72 h, these processes see increased gene participation, indicating an intensified effort to control viral replication as the infection advances. The “Type I interferon signaling pathway,” essential for antiviral defense, shows an increase in gene count from 4 at 24 h to 17 at 72 h, underscoring its growing importance in orchestrating the immune response over time.

Initially, the focus is on direct antiviral responses (“response to virus,” “viral genome replication”), which are relatively narrow in scope. As time progresses, there is a shift towards a broader engagement of the immune system, as seen in the increased gene counts in processes like “regulation of innate immune response” and “positive regulation of cytokine production,” reflecting a systemic activation of immune defenses.

The data reflects a typical immune response trajectory where the initial reaction involves rapid activation of antiviral genes and pathways that directly inhibit viral processes. Over time, as the infection persists or evolves, the immune system ramps up broader and more systemic processes, involving a larger set of genes and pathways that not only target the virus directly but also prepare the body for a sustained defensive effort. Many of the genes listed, such as IFIT1, MX1, and OASL, are well-known ISGs. Their increased participation from 24 to 72 h post-infection highlights the pivotal role of interferon-driven responses in shaping the antiviral defense over the course of a PIV3 infection.

In summary, the evolution of GO processes from 24 to 72 h post-infection underscores the dynamic nature of the host immune response to PIV3, characterized by an initial focus on direct antiviral actions which broadens into a comprehensive immune activation involving an array of cytokines and signaling pathways.

#### 4.5.6 Findings based on GO analysis

The GO analysis of common BH-significant transcripts across RNA-Seq and NanoString platforms has revealed substantial concordance in the identification and characterization of immune response genes following infections with IAV, MPV, and PIV3. This analysis underscores the robustness and reliability of both platforms in capturing biologically relevant changes across multiple time points and virus types.

For IAV and PIV3, both platforms consistently highlighted key processes such as “defense response to virus” and “response to virus” at both 24- and 72-h post-infection, demonstrating a sustained and evolving immune response. This included a notable activation of pathways like “negative regulation of viral process” and “type I interferon signaling pathway,” with significant contributions from genes such as IFIT1, MX1, RSAD2, and STAT1. The alignment of these results across both platforms not only reinforces the validity of the data but also highlights the dynamic and complex nature of the host defense mechanisms over the course of viral infections.

Moreover, the analysis detailed how specific pathways were intensified over time, particularly for IAV and PIV3, where an expansion in the number of genes involved in crucial immune processes was observed from 24 to 72 h. This transition from immediate antiviral responses to a broader, systemic immune engagement illustrates the platforms’ capability to accurately reflect the progression of the immune response to viral infections.

In contrast, the case of MPV demonstrated certain limitations of RNA-Seq in early detection, which were not observed with the NanoString platform. Despite this, by 72 h post-infection, some key genes were identified involved in critical immune processes, underscoring its utility in capturing significant gene expressions even when the initial immune response is subtle or slow to manifest.

This GO analysis not only confirms the technical consistency and biological relevance of the findings from both RNA-Seq and NanoString but also enhances our understanding of the molecular mechanisms underlying the response to viral infections. These insights are invaluable for developing targeted therapeutic strategies and for future research combining data from these robust platforms. The concordance observed here supports the complementary use of RNA-Seq and NanoString in comprehensive genomic studies, ensuring a deeper and more accurate exploration of gene expression dynamics in health and disease.

## 5 Conclusion

In this study, we conducted an extensive comparative analysis of RNA-Seq and NanoString technologies to assess gene expression in human lung organ-tissue equivalents (OTEs) during viral infections. Our investigation covered a substantial spectrum of 19,671 genes through RNA-Seq and 773 immune-related genes via NanoString, focusing on their expression in the context of Influenza A virus (IAV), Human metapneumovirus (MPV), and Parainfluenza virus 3 (PIV3) under various infection scenarios including UV-inactivated and active viral states. Our analysis employed various methods such as Spearman correlation, Distance correlation, Bland-Altman analysis, GLMs, Huber regression, the Magnitude-Altitude Score (MAS) algorithm, and Gene Ontology (GO) analysis to compare the RNA-Seq and NanoString data comprehensively. The MAS algorithm, which integrates both the amplitude of gene expression changes (magnitude) and their statistical relevance (altitude), provides a holistic method to rank genes according to their distinct expression patterns during particular viral infection conditions.

The correlation analysis, employing Spearman and Distance correlation methods, conclusively demonstrates that both RNA-Seq and NanoString are robust and reliable platforms for gene expression analysis. These methods showed strong correlations, consistently ranging from 0.86 to 0.90, across various experimental setups and time points in an OTE model.

The Bland-Altman analysis conducted across various infection conditions and time points substantiates a high level of agreement between RNA-Seq and NanoString platforms, with the vast majority of gene expression measurements falling within the agreed limits of concordance. Specifically, over 96.6% of genes for the paired measurements in each condition are within the limits, indicating minimal bias and confirming the reliability of these methods for comparative gene expression studies.

The comprehensive analysis utilizing GLMs, and Huber regression has effectively demonstrated a high degree of agreement between RNA-Seq and NanoString platforms across various infection conditions. Both analytical approaches reveal significant consistency, with high Pseudo $R^{2}$ values indicating strong model fits and minimal variation unexplained by the models. The remarkably low *p*-values across all conditions confirm the statistical significance of the relationship between the datasets.

The Concordance Analysis using the Magnitude-Altitude Score (MAS) algorithm effectively demonstrated the agreement and discrepancies between RNA-Seq and NanoString datasets across a variety of infection conditions. A robust alignment between the platforms was evident, particularly in the identification of crucial antiviral defense genes. Genes like ISG15, MX1, RSAD2, as well as members of the OAS family (OAS1, OAS2, OAS3, OASL), consistently emerged as key players. The IFIT proteins (IFIT1, IFIT2, IFIT3) were highlighted for their role in countering viral replication, while CXCL10 and CXCL11 shed light on the OTEs’ innate immune response against viral challenges.

The GO analysis of common BH-significant transcripts across RNA-Seq and NanoString platforms has revealed substantial concordance in the identification and characterization of immune response genes following infections with IAV, MPV, and PIV3. This analysis underscores the robustness and reliability of both platforms in capturing biologically relevant changes across multiple time points and virus types. For IAV and PIV3, both platforms consistently highlighted key processes such as “defense response to virus” and “response to virus” at both 24- and 72-h post-infection, demonstrating a sustained and evolving immune response. This included a notable activation of pathways like “negative regulation of viral process” and “type I interferon signaling pathway,” with significant contributions from genes such as IFIT1, MX1, RSAD2, and STAT1. The alignment of these results across both platforms not only reinforces the validity of the data but also highlights the dynamic and complex nature of the host defense mechanisms over the course of viral infections. Moreover, the analysis detailed how specific pathways were intensified over time, particularly for IAV and PIV3, where an expansion in the number of genes involved in crucial immune processes was observed from 24 to 72 h. This transition from immediate antiviral responses to a broader, systemic immune engagement illustrates the platforms’ capability to accurately reflect the progression of the immune response to viral infections.

In summary, the study demonstrated a high level of agreement between RNA-Seq and NanoString technologies in analyzing gene expression within OTEs during viral infections.

## 6 Limitations of the study

• Sensitivity differences between platforms: While both RNA-Seq and NanoString were generally consistent, discrepancies in sensitivity were observed, particularly in the early detection of viral responses, as noted with the MPV infection. This suggests that each platform may have unique strengths and limitations that could influence the detection and analysis of low-abundance transcripts or subtle gene expression changes.
• Inherent technological biases: Each platform comes with inherent biases, such as the normalization techniques and data processing strategies, which could affect the interpretation of results. For example, different normalization methods used in RNA-Seq and NanoString might have contributed to the observed discrepancies and trends in the Bland-Altman plots.
• Scope of gene analysis: The study focused on a set of common genes identified across both platforms, which might not represent the entire transcriptomic landscape. Important regulatory or low-abundance genes uniquely detected by only one platform might have been overlooked, potentially omitting significant biological insights.
• Generalizability of findings: The conclusions drawn from this study are based on a specific 3D airway OTE model and certain viral infections. Extending these findings to other models or infections might require additional validation to ensure that the observed gene expression dynamics and immune responses are broadly applicable.

## Data Availability

The data analyzed in this study is subject to the following licenses/restrictions: The data cannot be shared publicly because it is owned by the U.S. Government. However, the data are available from the Wake Forest Institute for Regenerative Medicine for researchers who meet the criteria for access to the data. In that case, the data underlying this article will be shared upon reasonable request to the corresponding author. Requests to access these datasets should be directed to mrezapou@wakehealth.edu.

## References

[B1] Benjamini Y. Heller R. Yekutieli D. (2009). Selective inference in complex research. Philosophical Trans. R. Soc. A Math. Phys. Eng. Sci. 367, 4255–4271. 10.1098/rsta.2009.0127 PMC3263782 19805444

[B2] Benjamini Y. Hochberg Y. (1995). Controlling the false discovery rate: a practical and powerful approach to multiple testing. J. R. Stat. Soc. Ser. B Methodol. 57 (1), 289–300. 10.1111/j.2517-6161.1995.tb02031.x

[B3] Carlson M. Falcon S. Pages H. Li N. (2019). And others, "org. Hs. eg. db: genome wide annotation for Human. R. package version 3 (2), 3.

[B4] Dismuke C. Lindrooth R. (2006). Ordinary least squares. Methods Des. outcomes Res. 93 (1), 93–104.

[B5] Geiss G. K. Bumgarner R. E. Birditt B. Dahl T. Dowidar N. Dunaway L. (2008). Direct multiplexed measurement of gene expression with color-coded probe pairs," Nat. Biotechnol. 26 (3), 317–325. 10.1038/nbt1385 18278033

[B6] Giavarina D. (2015). Understanding bland altman analysis. Biochem. medica 25 (2), 141–151. 10.11613/BM.2015.015 PMC4470095 26110027

[B7] Hauke J. Kossowski T. (2011). Comparison of values of Pearson's and Spearman's correlation coefficients on the same sets of data. Quaest. Geogr. 30 (2), 87–93. 10.2478/v10117-011-0021-1

[B8] Huber P. J. (1996) Robust statistical procedures. Philadelphia, PA: SIAM.

[B9] Law C. W. Chen Y. Shi W. Smyth G. K. (2014). voom: precision weights unlock linear model analysis tools for RNA-seq read counts. Genome Biol. 15 (2), R29–R17. 10.1186/gb-2014-15-2-r29 24485249 PMC4053721

[B10] Leach T. Gandhi U. Reeves Stumpf K. Okuda K. Marini (2023). Development of a novel air-liquid interface airway tissue equivalent model for *in vitro* respiratory modeling studies. Sci. Rep. 13 (1), 10137. 10.1038/s41598-023-36863-1 37349353 PMC10287689

[B11] Love M. I. Huber W. Anders S. (2014). Moderated estimation of fold change and dispersion for RNA-seq data with DESeq2. Genome Biol. 15, 1–21. 10.1186/s13059-014-0550-8 PMC4302049 25516281

[B12] Maronna R. A. Martin R. D. Yohai V. J. Salibián-Barrera M. (2019) Robust statistics: theory and methods (with R). John Wiley & Sons.

[B13] Martin M. (2011). Cutadapt removes adapter sequences from high-throughput sequencing reads. EMBnet. J. 17 (1), 10–12. 10.14806/ej.17.1.200

[B14] Nelder J. A. Wedderburn R. W. M. (1972). Generalized linear models. J. R. Stat. Soc. Ser. A General. 135 (3), 370–384. 10.2307/2344614

[B15] Rezapour M. Walker S. J. Ornelles D. A. McNutt P. M. Gurcan Nafi M. (2024). Analysis of gene expression dynamics and differential expression in viral infections using generalized linear models and quasi-likelihood methods. Front. Microbiol. 15, 1342328. 10.3389/fmicb.2024.1342328 38655085 PMC11037428

[B16] Székely G. J. Rizzo M. L. (2009). Brownian distance covariance. Ann. Appl. statistics 3, 1236–1265. 10.1214/09-aoas312 PMC2889501 20574547

[B17] Wang Z. Gerstein M. Snyder M. (2009). RNA-Seq: a revolutionary tool for transcriptomics. Nat. Rev. Genet. 10 (1), 57–63. 10.1038/nrg2484 19015660 PMC2949280

[B18] Wedderburn R. W. (1974). Quasi-likelihood functions, generalized linear models, and the Gauss—Newton method. Biometrika 61 (3), 439–447. 10.2307/2334725

[B19] Wu T. Hu E. Xu S. Chen M. Guo P. Dai (2021). clusterProfiler 4.0: a universal enrichment tool for interpreting omics data. innovation 2 (3), 100141. 10.1016/j.xinn.2021.100141 34557778 PMC8454663

[B20] Xing Yi Yu T. Wu Nian Y. Roy M. Kim J. (2006). An expectation-maximization algorithm for probabilistic reconstructions of full-length isoforms from splice graphs. Nucleic acids Res. 34 (10), 3150–3160. 10.1093/nar/gkl396 16757580 PMC1475746

[B21] Young M. D. Wakefield M. J. Smyth G. K. Oshlack A. (2010). Ene ontology analysis for RNA-seq: accounting for selection bias. Genome Biol. 11, 1–12. 10.1186/gb-2010-11-2-r14 PMC2872874 20132535

