# Supplementary material for: A comparative analysis of RNA-Seq and NanoString technologies in deciphering viral infection response in upper airway lung organoids
Source: Front Genet. 2024 Jun 18;15:1327984. doi: 10.3389/fgene.2024.1327984 (PMC11217172; doi:10.3389/fgene.2024.1327984)
Supplement: Supplementary file 1 [file Table1.DOCX]

Supplementary Materials for

A Comparative Analysis of RNA-Seq and NanoString Technologies in Deciphering Viral Infection Response in Upper Airway Lung Organoids

Mostafa Rezapour, PhD^1*^; Stephen J. Walker, PhD ^2^; David A. Ornelles, PhD ^3^; M. Khalid Khan Niazi, PhD ^1^; Patrick M. McNutt, PhD ^2^; Anthony Atala, MD ^2^; Metin Nafi Gurcan, PhD ^1^

^1^Center for Artificial Intelligence Research, Wake Forest University School of Medicine, Winston-Salem, NC, USA

^2^ Wake Forest Institute for Regenerative Medicine, Wake Forest University School of Medicine, Winston-Salem, NC, USA

^3^ Department of Microbiology and Immunology, Wake Forest University School of Medicine, Winston-Salem, NC, USA

*** Correspondence:**Mostafa Rezapour, PhD
[mrezapou@wakehealth.edu](mailto:mrezapou@wakehealth.edu)

# **Tables**

**Table S1** displays 16 different infection conditions that are classified based on (1) Virus, (2) Treat, and (3) post-infection time. In this study, OTEs are infected by three viruses: IAV, MPV, and PIV3; there are two kinds of treatments: UV and Non-UV (or active); and there are two post-infection time points, 24-hours, and 72-hours. **Table S2** describes the distribution of data points between post-infection time points, experimental groups, negative control groups, and control groups.

**Table S1.** Sixteen infection conditions classified based on (1) Virus, (2) Treat, and (3) post-infection time.

| **Condition** | **Virus** | **Treat** | **Post-infection Time** | **Condition** | **Virus** | **Treat** | **Post-infection Time** |
| --- | --- | --- | --- | --- | --- | --- | --- |
| IAV-UV-24 | IAV | UV | 24-hours | IAV-UV-72 | IAV | UV | 72-hours |
| IAV-None-24 | IAV | Non-UV | 24-hours | IAV-None-72 | IAV | Non-UV | 72-hours |
| MPV-UV-24 | MPV | UV | 24-hours | MPV-UV-72 | MPV | UV | 72-hours |
| MPV-None-24 | MPV | Non-UV | 24-hours | MPV-None-72 | MPV | Non-UV | 72-hours |
| PIV3-UV-24 | PIV3 | UV | 24-hours | PIV3-UV-72 | PIV3 | UV | 72-hours |
| PIV3-None-24 | PIV3 | Non-UV | 24-hours | PIV3-None-72 | PIV3 | Non-UV | 72-hours |
| Mock-24 | Mock | - | 24-hours | Mock-72 | Mock | - | 72-hours |
| Naïve-24 | Untreated | - | 24-hours | Naïve-72 | Untreated | - | 72-hours |

**Table S2.** Distribution of replicates between post-infection timepoints, experimental groups, negative and regular control groups.

| At post-infection time point **24-hours** | **6 replicates for each of the followings:**  **Experimental Group:** Non-UV treated IAV, Non-UV treated MPV, Non-UV treated PIV3  **Negative Control Group:** UV treated IAV, UV treated MPV, UV treated PIV3  **Control Group:** Mock, Naïve |
| --- | --- |
| At post-infection time point **72-hours** | **6 replicates for each of the followings:**  **Experimental Group:** Non-UV treated IAV, Non-UV treated MPV, Non-UV treated PIV3  **Negative Control Group:** UV treated IAV, UV treated MPV, UV treated PIV3  **Control Group:** Mock, Naïve |

**Table S3. Top 20 Significant GO Biological Processes of 2023 for Upregulated, Significant Common Genes Between RNA-Seq and NanoString When Comparing IAV-None-24 against Mock-24.**

| **ID** | **Description** | **qvalue** | **Count** | **Gene Symbols** |
| --- | --- | --- | --- | --- |
| GO:0009615 | response to virus | 8.16E-53 | 45 | IFIT2, RSAD2, IFIT3, IFNL1, IFIT1, IFNB1, ISG15, OAS2, OASL, MX1, HERC5, OAS1, OAS3, IFI44, DDX58, IFIH1, CXCL10, CCL5, IFI6, IFITM1, EIF2AK2, IFI16, STAT1, IRF1, ZBP1, GBP1, TNF, DTX3L, IFITM2, SAMHD1, APOBEC3G, AIM2, TLR3, TRIM5, PARP9, IFITM3, MLKL, IL12A, CGAS, IFI27, BST2, TRIM22, MYD88, IL15, CXCL9 |
| GO:0051607 | defense response to virus | 3.47E-52 | 41 | IFIT2, RSAD2, IFIT3, IFNL1, IFIT1, IFNB1, ISG15, OAS2, OASL, MX1, HERC5, OAS1, OAS3, DDX58, IFIH1, CXCL10, IFI6, IFITM1, EIF2AK2, IFI16, STAT1, IRF1, ZBP1, GBP1, DTX3L, IFITM2, SAMHD1, APOBEC3G, AIM2, TLR3, TRIM5, PARP9, IFITM3, MLKL, CGAS, IFI27, BST2, TRIM22, MYD88, IL15, CXCL9 |
| GO:0140546 | defense response to symbiont | 3.47E-52 | 41 | IFIT2, RSAD2, IFIT3, IFNL1, IFIT1, IFNB1, ISG15, OAS2, OASL, MX1, HERC5, OAS1, OAS3, DDX58, IFIH1, CXCL10, IFI6, IFITM1, EIF2AK2, IFI16, STAT1, IRF1, ZBP1, GBP1, DTX3L, IFITM2, SAMHD1, APOBEC3G, AIM2, TLR3, TRIM5, PARP9, IFITM3, MLKL, CGAS, IFI27, BST2, TRIM22, MYD88, IL15, CXCL9 |
| GO:0048525 | negative regulation of viral process | 9.99E-33 | 23 | RSAD2, IFIT1, IFNB1, ISG15, OAS2, OASL, MX1, OAS1, OAS3, IFIH1, CCL5, TRIM21, IFITM1, EIF2AK2, IFI16, STAT1, TNF, IFITM2, APOBEC3G, TRIM5, IFITM3, BST2, TRIM25 |
| GO:0050792 | regulation of viral process | 4.87E-30 | 25 | RSAD2, IFIT1, IFNB1, ISG15, OAS2, OASL, MX1, OAS1, OAS3, IFIH1, CCL5, LAMP3, TRIM21, IFITM1, EIF2AK2, IFI16, STAT1, TNF, IFITM2, APOBEC3G, TRIM5, IFITM3, BST2, TRIM22, TRIM25 |
| GO:0045071 | negative regulation of viral genome replication | 1.00E-29 | 19 | RSAD2, IFIT1, IFNB1, ISG15, OAS2, OASL, MX1, OAS1, OAS3, IFIH1, CCL5, IFITM1, EIF2AK2, IFI16, TNF, IFITM2, APOBEC3G, IFITM3, BST2 |
| GO:1903900 | regulation of viral life cycle | 1.47E-29 | 24 | RSAD2, IFIT1, IFNB1, ISG15, OAS2, OASL, MX1, OAS1, OAS3, IFIH1, CCL5, LAMP3, TRIM21, IFITM1, EIF2AK2, IFI16, TNF, IFITM2, APOBEC3G, TRIM5, IFITM3, BST2, TRIM22, TRIM25 |
| GO:0019221 | cytokine-mediated signaling pathway | 1.67E-29 | 33 | IFNB1, ISG15, OAS2, OASL, MX1, OAS1, OAS3, CXCL10, CCL5, IFITM1, CXCL11, STAT1, TNFSF13B, IRF1, ZBP1, TNF, SOCS1, SP100, IFITM2, SAMHD1, AIM2, PARP9, IFITM3, IFI27, CASP1, JAK2, MYD88, FAS, IL15, IL15RA, IL1R2, CXCL9, CX3CL1 |
| GO:0002831 | regulation of response to biotic stimulus | 1.55E-28 | 29 | IFIT1, IFNB1, ISG15, OASL, HERC5, OAS1, OAS3, DDX58, CCL5, CD274, TRIM21, IFI35, IFI16, STAT1, IRF1, ZBP1, DTX3L, SOCS1, LAG3, SAMHD1, APOBEC3G, GBP5, AIM2, TRIM5, PARP9, IL12A, CGAS, IL15, CX3CL1 |
| GO:0045069 | regulation of viral genome replication | 5.51E-26 | 19 | RSAD2, IFIT1, IFNB1, ISG15, OAS2, OASL, MX1, OAS1, OAS3, IFIH1, CCL5, IFITM1, EIF2AK2, IFI16, TNF, IFITM2, APOBEC3G, IFITM3, BST2 |
| GO:0019079 | viral genome replication | 6.72E-24 | 20 | RSAD2, IFIT1, IFNB1, ISG15, OAS2, OASL, MX1, OAS1, OAS3, IFIH1, CCL5, IFITM1, EIF2AK2, IFI16, TNF, IFITM2, APOBEC3G, IFITM3, IFI27, BST2 |
| GO:0034340 | response to type I interferon | 2.31E-23 | 16 | IFIT1, IFNB1, ISG15, OAS2, MX1, OAS1, OAS3, IFITM1, STAT1, ZBP1, SP100, IFITM2, SAMHD1, IFITM3, IFI27, MYD88 |
| GO:0019058 | viral life cycle | 4.46E-23 | 25 | RSAD2, IFIT1, IFNB1, ISG15, OAS2, OASL, MX1, OAS1, OAS3, IFIH1, CCL5, LAMP3, TRIM21, IFITM1, EIF2AK2, IFI16, TNF, IFITM2, APOBEC3G, TRIM5, IFITM3, IFI27, BST2, TRIM22, TRIM25 |
| GO:0016032 | viral process | 6.56E-23 | 27 | RSAD2, IFIT1, IFNB1, ISG15, OAS2, OASL, MX1, OAS1, OAS3, IFIH1, CCL5, LAMP3, TRIM21, IFITM1, EIF2AK2, IFI16, STAT1, TNF, IFITM2, APOBEC3G, TRIM5, PARP9, IFITM3, IFI27, BST2, TRIM22, TRIM25 |
| GO:0071357 | cellular response to type I interferon | 2.98E-22 | 15 | IFIT1, IFNB1, ISG15, OAS2, OAS1, OAS3, IFITM1, STAT1, ZBP1, SP100, IFITM2, SAMHD1, IFITM3, IFI27, MYD88 |
| GO:0060337 | type I interferon signaling pathway | 1.64E-20 | 14 | IFNB1, ISG15, OAS2, OAS1, OAS3, IFITM1, STAT1, ZBP1, SP100, IFITM2, SAMHD1, IFITM3, IFI27, MYD88 |
| GO:0034341 | response to interferon-gamma | 4.59E-20 | 18 | CCL5, GBP4, TRIM21, IFITM1, STAT1, IRF1, GBP1, SOCS1, SP100, IFITM2, GBP5, TLR3, PARP9, IFITM3, BST2, CASP1, JAK2, CX3CL1 |
| GO:0035456 | response to interferon-beta | 2.76E-19 | 12 | IFNB1, OAS1, IFITM1, IFI16, STAT1, IRF1, XAF1, IFITM2, AIM2, TLR3, IFITM3, BST2 |
| GO:0001819 | positive regulation of cytokine production | 4.34E-19 | 25 | RSAD2, IFNL1, ISG15, OAS2, OAS1, OAS3, DDX58, IFIH1, CD274, EIF2AK2, IFI16, STAT1, IRF1, TNF, GBP5, AIM2, TLR3, IL12A, CGAS, CASP1, JAK2, MYD88, IL15, PTGER4, CX3CL1 |
| GO:0045088 | regulation of innate immune response | 4.20E-18 | 19 | IFNB1, ISG15, OAS1, OAS3, CCL5, TRIM21, IFI35, IFI16, IRF1, ZBP1, SOCS1, LAG3, SAMHD1, GBP5, AIM2, TRIM5, PARP9, IL12A, CGAS |

**Table S4. Top 20 Significant GO Biological Processes of 2023 for Upregulated, Significant Common Genes Between RNA-Seq and NanoString When Comparing PIV3-None-24 against Mock-24.**

| **ID** | **Description** | **qvalue** | **Count** | **Gene Symbols** |
| --- | --- | --- | --- | --- |
| GO:0051607 | defense response to virus | 1.63E-23 | 14 | IFIT1, CXCL10, IFIT3, IFIT2, MX1, RSAD2, HERC5, OAS2, ISG15, OAS3, DDX58, EIF2AK2, OASL, PARP9 |
| GO:0140546 | defense response to symbiont | 1.63E-23 | 14 | IFIT1, CXCL10, IFIT3, IFIT2, MX1, RSAD2, HERC5, OAS2, ISG15, OAS3, DDX58, EIF2AK2, OASL, PARP9 |
| GO:0009615 | response to virus | 1.14E-21 | 14 | IFIT1, CXCL10, IFIT3, IFIT2, MX1, RSAD2, HERC5, OAS2, ISG15, OAS3, DDX58, EIF2AK2, OASL, PARP9 |
| GO:0045071 | negative regulation of viral genome replication | 1.44E-15 | 8 | IFIT1, MX1, RSAD2, OAS2, ISG15, OAS3, EIF2AK2, OASL |
| GO:0045069 | regulation of viral genome replication | 3.87E-14 | 8 | IFIT1, MX1, RSAD2, OAS2, ISG15, OAS3, EIF2AK2, OASL |
| GO:0048525 | negative regulation of viral process | 6.23E-14 | 8 | IFIT1, MX1, RSAD2, OAS2, ISG15, OAS3, EIF2AK2, OASL |
| GO:0019079 | viral genome replication | 9.80E-13 | 8 | IFIT1, MX1, RSAD2, OAS2, ISG15, OAS3, EIF2AK2, OASL |
| GO:1903900 | regulation of viral life cycle | 2.32E-12 | 8 | IFIT1, MX1, RSAD2, OAS2, ISG15, OAS3, EIF2AK2, OASL |
| GO:0050792 | regulation of viral process | 4.75E-12 | 8 | IFIT1, MX1, RSAD2, OAS2, ISG15, OAS3, EIF2AK2, OASL |
| GO:0016032 | viral process | 1.27E-10 | 9 | IFIT1, MX1, RSAD2, OAS2, ISG15, OAS3, EIF2AK2, OASL, PARP9 |
| GO:0019058 | viral life cycle | 7.83E-10 | 8 | IFIT1, MX1, RSAD2, OAS2, ISG15, OAS3, EIF2AK2, OASL |
| GO:0140374 | antiviral innate immune response | 7.24E-09 | 4 | IFIT1, CXCL10, MX1, DDX58 |
| GO:0034340 | response to type I interferon | 1.29E-08 | 5 | IFIT1, MX1, OAS2, ISG15, OAS3 |
| GO:0002831 | regulation of response to biotic stimulus | 4.55E-08 | 7 | IFIT1, HERC5, ISG15, OAS3, DDX58, OASL, PARP9 |
| GO:0032728 | positive regulation of interferon-beta production | 3.45E-07 | 4 | OAS2, ISG15, OAS3, DDX58 |
| GO:0019221 | cytokine-mediated signaling pathway | 5.02E-07 | 7 | CXCL10, MX1, OAS2, ISG15, OAS3, OASL, PARP9 |
| GO:0071357 | cellular response to type I interferon | 9.96E-07 | 4 | IFIT1, OAS2, ISG15, OAS3 |
| GO:0032608 | interferon-beta production | 1.21E-06 | 4 | OAS2, ISG15, OAS3, DDX58 |
| GO:0032648 | regulation of interferon-beta production | 1.21E-06 | 4 | OAS2, ISG15, OAS3, DDX58 |
| GO:0032481 | positive regulation of type I interferon production | 1.32E-06 | 4 | OAS2, ISG15, OAS3, DDX58 |

**Table S5. Top 20 Significant GO Biological Processes of 2023 for Upregulated, Significant Common Genes Between RNA-Seq and NanoString When Comparing IAV-None-24 against Mock-24.**

| ID | Description | qvalue | Count | GeneSymbols |
| --- | --- | --- | --- | --- |
| GO:0051607 | defense response to virus | 2.32E-54 | 43 | IFIT1, CXCL10, RSAD2, IFITM1, IFIT3, OAS2, IFIT2, OAS1, MX1, OAS3, OASL, ISG15, HERC5, DDX58, IFNL1, IFIH1, IFI6, STAT1, BST2, IFITM3, IFI27, IFITM2, EIF2AK2, GBP1, PARP9, IRF7, SAMHD1, IFI16, DHX58, DTX3L, IFNB1, AIM2, IRF1, ADAR, TLR2, STAT2, TRIM22, APOBEC3G, ZBP1, TRIM5, MLKL, IL15, MYD88 |
| GO:0140546 | defense response to symbiont | 2.32E-54 | 43 | IFIT1, CXCL10, RSAD2, IFITM1, IFIT3, OAS2, IFIT2, OAS1, MX1, OAS3, OASL, ISG15, HERC5, DDX58, IFNL1, IFIH1, IFI6, STAT1, BST2, IFITM3, IFI27, IFITM2, EIF2AK2, GBP1, PARP9, IRF7, SAMHD1, IFI16, DHX58, DTX3L, IFNB1, AIM2, IRF1, ADAR, TLR2, STAT2, TRIM22, APOBEC3G, ZBP1, TRIM5, MLKL, IL15, MYD88 |
| GO:0009615 | response to virus | 1.87E-53 | 46 | IFIT1, CXCL10, RSAD2, IFITM1, IFIT3, IFI44, OAS2, IFIT2, OAS1, CCL5, MX1, OAS3, OASL, ISG15, HERC5, DDX58, IFNL1, IFIH1, IFI6, STAT1, BST2, IFITM3, IFI27, IFITM2, EIF2AK2, GBP1, PARP9, IRF7, SAMHD1, IFI16, DHX58, DTX3L, IFNB1, AIM2, IRF1, ADAR, TNF, TLR2, STAT2, TRIM22, APOBEC3G, ZBP1, TRIM5, MLKL, IL15, MYD88 |
| GO:0002831 | regulation of response to biotic stimulus | 1.07E-36 | 35 | IFIT1, OAS1, CCL5, OAS3, OASL, ISG15, HERC5, DDX58, STAT1, IFI35, CD274, TRIM21, PARP9, IRF7, HLA-B, SAMHD1, IFI16, DHX58, DTX3L, LAG3, IFNB1, GBP5, AIM2, IRF1, ADAR, STAT2, APOBEC3G, ZBP1, TRIM5, HLA-E, HLA-A, NCF1, NOD2, SOCS1, IL15 |
| GO:0048525 | negative regulation of viral process | 3.99E-32 | 23 | IFIT1, RSAD2, IFITM1, OAS2, OAS1, CCL5, MX1, OAS3, OASL, ISG15, IFIH1, STAT1, BST2, IFITM3, IFITM2, EIF2AK2, TRIM21, IFI16, IFNB1, TNF, APOBEC3G, TRIM5, TRIM25 |
| GO:0050792 | regulation of viral process | 4.14E-31 | 26 | IFIT1, RSAD2, IFITM1, OAS2, OAS1, CCL5, MX1, OAS3, OASL, ISG15, IFIH1, LAMP3, STAT1, BST2, IFITM3, IFITM2, EIF2AK2, TRIM21, IFI16, IFNB1, ADAR, TNF, TRIM22, APOBEC3G, TRIM5, TRIM25 |
| GO:1903900 | regulation of viral life cycle | 1.31E-30 | 25 | IFIT1, RSAD2, IFITM1, OAS2, OAS1, CCL5, MX1, OAS3, OASL, ISG15, IFIH1, LAMP3, BST2, IFITM3, IFITM2, EIF2AK2, TRIM21, IFI16, IFNB1, ADAR, TNF, TRIM22, APOBEC3G, TRIM5, TRIM25 |
| GO:0019221 | cytokine-mediated signaling pathway | 6.94E-30 | 34 | CXCL10, IFITM1, OAS2, OAS1, CCL5, MX1, OAS3, OASL, ISG15, CXCL11, STAT1, IFITM3, IFI27, IFITM2, TNFSF13B, PARP9, IRF7, SP100, SAMHD1, IFNB1, AIM2, IRF1, ADAR, TNF, STAT2, ZBP1, CASP1, CSF1, SOCS1, IL15, MYD88, CCL2, IL2RG, IL15RA |
| GO:0045071 | negative regulation of viral genome replication | 2.46E-29 | 19 | IFIT1, RSAD2, IFITM1, OAS2, OAS1, CCL5, MX1, OAS3, OASL, ISG15, IFIH1, BST2, IFITM3, IFITM2, EIF2AK2, IFI16, IFNB1, TNF, APOBEC3G |
| GO:0034340 | response to type I interferon | 4.90E-29 | 19 | IFIT1, IFITM1, OAS2, OAS1, MX1, OAS3, ISG15, STAT1, IFITM3, IFI27, IFITM2, IRF7, SP100, SAMHD1, IFNB1, ADAR, STAT2, ZBP1, MYD88 |
| GO:0045088 | regulation of innate immune response | 5.14E-28 | 26 | OAS1, CCL5, OAS3, ISG15, IFI35, TRIM21, PARP9, IRF7, HLA-B, SAMHD1, IFI16, DHX58, LAG3, IFNB1, GBP5, AIM2, IRF1, ADAR, STAT2, ZBP1, TRIM5, HLA-E, HLA-A, NCF1, NOD2, SOCS1 |
| GO:0071357 | cellular response to type I interferon | 5.14E-28 | 18 | IFIT1, IFITM1, OAS2, OAS1, OAS3, ISG15, STAT1, IFITM3, IFI27, IFITM2, IRF7, SP100, SAMHD1, IFNB1, ADAR, STAT2, ZBP1, MYD88 |
| GO:0045069 | regulation of viral genome replication | 1.84E-27 | 20 | IFIT1, RSAD2, IFITM1, OAS2, OAS1, CCL5, MX1, OAS3, OASL, ISG15, IFIH1, BST2, IFITM3, IFITM2, EIF2AK2, IFI16, IFNB1, ADAR, TNF, APOBEC3G |
| GO:0019079 | viral genome replication | 6.76E-27 | 22 | IFIT1, RSAD2, IFITM1, OAS2, OAS1, CCL5, MX1, OAS3, OASL, ISG15, IFIH1, BST2, IFITM3, IFI27, IFITM2, EIF2AK2, IFI16, IFNB1, ADAR, TNF, APOBEC3G, CCL2 |
| GO:0060337 | type I interferon signaling pathway | 2.58E-26 | 17 | IFITM1, OAS2, OAS1, OAS3, ISG15, STAT1, IFITM3, IFI27, IFITM2, IRF7, SP100, SAMHD1, IFNB1, ADAR, STAT2, ZBP1, MYD88 |
| GO:0016032 | viral process | 2.81E-26 | 30 | IFIT1, RSAD2, IFITM1, OAS2, OAS1, CCL5, MX1, OAS3, OASL, ISG15, IFIH1, LAMP3, STAT1, BST2, IFITM3, IFI27, IFITM2, EIF2AK2, TRIM21, PARP9, IRF7, IFI16, IFNB1, ADAR, TNF, TRIM22, APOBEC3G, TRIM5, TRIM25, CCL2 |
| GO:0019058 | viral life cycle | 2.49E-25 | 27 | IFIT1, RSAD2, IFITM1, OAS2, OAS1, CCL5, MX1, OAS3, OASL, ISG15, IFIH1, LAMP3, BST2, IFITM3, IFI27, IFITM2, EIF2AK2, TRIM21, IFI16, IFNB1, ADAR, TNF, TRIM22, APOBEC3G, TRIM5, TRIM25, CCL2 |
| GO:0002683 | negative regulation of immune system process | 4.15E-19 | 25 | OAS1, OAS3, ISG15, IFNL1, BST2, CD274, TRIM21, GBP1, HLA-B, SAMHD1, IFI16, DHX58, LAG3, IFNB1, IRF1, ADAR, TNF, STAT2, CD68, HLA-E, HLA-A, NOD2, SOCS1, MAFB, CCL2 |
| GO:0001819 | positive regulation of cytokine production | 2.33E-18 | 25 | RSAD2, OAS2, OAS1, OAS3, ISG15, DDX58, IFNL1, IFIH1, STAT1, EIF2AK2, CD274, IRF7, IFI16, DHX58, GBP5, AIM2, IRF1, TNF, TLR2, CASP1, HLA-E, HLA-A, NOD2, IL15, MYD88 |
| GO:0034341 | response to interferon-gamma | 5.06E-18 | 17 | IFITM1, CCL5, STAT1, BST2, IFITM3, GBP4, IFITM2, TRIM21, GBP1, PARP9, SP100, GBP5, IRF1, TLR2, CASP1, SOCS1, CCL2 |

**Table S6. Top 20 Significant GO Biological Processes of 2023 for Upregulated, Significant Common Genes Between RNA-Seq and NanoString When Comparing MPV-None-72 against Mock-72.**

| **ID** | **Description** | **qvalue** | **Count** | **Gene Symbols** |
| --- | --- | --- | --- | --- |
| GO:0009615 | response to virus | 5.34E-08 | 5 | IFI44, OASL, OAS3, IRF9, IRF7 |
| GO:0039528 | cytoplasmic pattern recognition receptor signaling pathway in response to virus | 5.17E-07 | 3 | OASL, OAS3, IRF7 |
| GO:0051607 | defense response to virus | 9.19E-07 | 4 | OASL, OAS3, IRF9, IRF7 |
| GO:0140546 | defense response to symbiont | 9.19E-07 | 4 | OASL, OAS3, IRF9, IRF7 |
| GO:0002753 | cytoplasmic pattern recognition receptor signaling pathway | 1.18E-06 | 3 | OASL, OAS3, IRF7 |
| GO:0098586 | cellular response to virus | 2.73E-06 | 3 | OASL, OAS3, IRF7 |
| GO:0039530 | MDA-5 signaling pathway | 8.49E-06 | 2 | OAS3, IRF7 |
| GO:0002221 | pattern recognition receptor signaling pathway | 1.78E-05 | 3 | OASL, OAS3, IRF7 |
| GO:0032069 | regulation of nuclease activity | 2.77E-05 | 2 | OASL, OAS3 |
| GO:0039529 | RIG-I signaling pathway | 3.78E-05 | 2 | OASL, OAS3 |
| GO:0030522 | intracellular receptor signaling pathway | 4.73E-05 | 3 | OASL, OAS3, IRF7 |
| GO:0060338 | regulation of type I interferon-mediated signaling pathway | 5.65E-05 | 2 | OAS3, IRF7 |
| GO:0032728 | positive regulation of interferon-beta production | 6.14E-05 | 2 | OAS3, IRF7 |
| GO:0002831 | regulation of response to biotic stimulus | 6.96E-05 | 3 | OASL, OAS3, IRF7 |
| GO:0060337 | type I interferon signaling pathway | 8.46E-05 | 2 | OAS3, IRF7 |
| GO:0071357 | cellular response to type I interferon | 8.46E-05 | 2 | OAS3, IRF7 |
| GO:0032608 | interferon-beta production | 8.46E-05 | 2 | OAS3, IRF7 |
| GO:0032648 | regulation of interferon-beta production | 8.46E-05 | 2 | OAS3, IRF7 |
| GO:0045071 | negative regulation of viral genome replication | 8.46E-05 | 2 | OASL, OAS3 |
| GO:0032481 | positive regulation of type I interferon production | 8.46E-05 | 2 | OAS3, IRF7 |

**Table S7. Top 20 Significant GO Biological Processes of 2023 for Upregulated, Significant Common Genes Between RNA-Seq and NanoString When Comparing PIV3-None-72 against Mock-72.**

| ID | Description | qvalue | Count | GeneSymbols |
| --- | --- | --- | --- | --- |
| GO:0051607 | defense response to virus | 1.25E-59 | 45 | IFIT3, RSAD2, IFIT1, IFIT2, MX1, OAS3, OAS2, HERC5, DDX58, OAS1, CXCL10, IFIH1, IFITM1, STAT1, ISG15, IFNL1, IFI27, EIF2AK2, OASL, IFITM3, BST2, IFI6, GBP1, IFITM2, IFI16, SAMHD1, ZBP1, IFNB1, DTX3L, IRF7, TRIM22, PARP9, IRF1, DHX58, STAT2, ADAR, APOBEC3G, TLR3, IRF9, TRIM5, AIM2, MLKL, TLR2, IL15, MYD88 |
| GO:0140546 | defense response to symbiont | 1.25E-59 | 45 | IFIT3, RSAD2, IFIT1, IFIT2, MX1, OAS3, OAS2, HERC5, DDX58, OAS1, CXCL10, IFIH1, IFITM1, STAT1, ISG15, IFNL1, IFI27, EIF2AK2, OASL, IFITM3, BST2, IFI6, GBP1, IFITM2, IFI16, SAMHD1, ZBP1, IFNB1, DTX3L, IRF7, TRIM22, PARP9, IRF1, DHX58, STAT2, ADAR, APOBEC3G, TLR3, IRF9, TRIM5, AIM2, MLKL, TLR2, IL15, MYD88 |
| GO:0009615 | response to virus | 9.46E-57 | 47 | IFIT3, RSAD2, IFIT1, IFIT2, MX1, IFI44, OAS3, OAS2, HERC5, DDX58, OAS1, CXCL10, IFIH1, IFITM1, CCL5, STAT1, ISG15, IFNL1, IFI27, EIF2AK2, OASL, IFITM3, BST2, IFI6, GBP1, IFITM2, IFI16, SAMHD1, ZBP1, IFNB1, DTX3L, IRF7, TRIM22, PARP9, IRF1, DHX58, STAT2, ADAR, APOBEC3G, TLR3, IRF9, TRIM5, AIM2, MLKL, TLR2, IL15, MYD88 |
| GO:0002831 | regulation of response to biotic stimulus | 3.48E-31 | 31 | IFIT1, OAS3, HERC5, DDX58, OAS1, CCL5, STAT1, ISG15, IFI35, TRIM21, OASL, IFI16, SAMHD1, LAG3, CD274, ZBP1, IFNB1, DTX3L, IRF7, PARP9, IRF1, DHX58, STAT2, ADAR, GBP5, HLA-B, APOBEC3G, TRIM5, AIM2, HLA-E, IL15 |
| GO:0048525 | negative regulation of viral process | 9.50E-31 | 22 | RSAD2, IFIT1, MX1, OAS3, OAS2, OAS1, IFIH1, IFITM1, CCL5, STAT1, ISG15, EIF2AK2, TRIM21, OASL, IFITM3, BST2, IFITM2, IFI16, IFNB1, APOBEC3G, TRIM5, TRIM25 |
| GO:0050792 | regulation of viral process | 4.88E-30 | 25 | RSAD2, IFIT1, MX1, OAS3, OAS2, OAS1, IFIH1, IFITM1, CCL5, LAMP3, STAT1, ISG15, EIF2AK2, TRIM21, OASL, IFITM3, BST2, IFITM2, IFI16, IFNB1, TRIM22, ADAR, APOBEC3G, TRIM5, TRIM25 |
| GO:1903900 | regulation of viral life cycle | 1.74E-29 | 24 | RSAD2, IFIT1, MX1, OAS3, OAS2, OAS1, IFIH1, IFITM1, CCL5, LAMP3, ISG15, EIF2AK2, TRIM21, OASL, IFITM3, BST2, IFITM2, IFI16, IFNB1, TRIM22, ADAR, APOBEC3G, TRIM5, TRIM25 |
| GO:0034340 | response to type I interferon | 1.83E-29 | 19 | IFIT1, MX1, OAS3, OAS2, OAS1, IFITM1, STAT1, ISG15, SP100, IFI27, IFITM3, IFITM2, SAMHD1, ZBP1, IFNB1, IRF7, STAT2, ADAR, MYD88 |
| GO:0071357 | cellular response to type I interferon | 2.17E-28 | 18 | IFIT1, OAS3, OAS2, OAS1, IFITM1, STAT1, ISG15, SP100, IFI27, IFITM3, IFITM2, SAMHD1, ZBP1, IFNB1, IRF7, STAT2, ADAR, MYD88 |
| GO:0045071 | negative regulation of viral genome replication | 9.60E-28 | 18 | RSAD2, IFIT1, MX1, OAS3, OAS2, OAS1, IFIH1, IFITM1, CCL5, ISG15, EIF2AK2, OASL, IFITM3, BST2, IFITM2, IFI16, IFNB1, APOBEC3G |
| GO:0060337 | type I interferon signaling pathway | 1.18E-26 | 17 | OAS3, OAS2, OAS1, IFITM1, STAT1, ISG15, SP100, IFI27, IFITM3, IFITM2, SAMHD1, ZBP1, IFNB1, IRF7, STAT2, ADAR, MYD88 |
| GO:0045069 | regulation of viral genome replication | 5.02E-26 | 19 | RSAD2, IFIT1, MX1, OAS3, OAS2, OAS1, IFIH1, IFITM1, CCL5, ISG15, EIF2AK2, OASL, IFITM3, BST2, IFITM2, IFI16, IFNB1, ADAR, APOBEC3G |
| GO:0019221 | cytokine-mediated signaling pathway | 2.47E-25 | 30 | MX1, OAS3, OAS2, OAS1, CXCL10, IFITM1, CCL5, CXCL11, STAT1, ISG15, SP100, IFI27, OASL, IFITM3, IFITM2, SAMHD1, ZBP1, IFNB1, TNFSF13B, IRF7, PARP9, IRF1, STAT2, ADAR, CASP1, AIM2, JAK2, IL15, ACKR4, MYD88 |
| GO:0016032 | viral process | 3.37E-24 | 28 | RSAD2, IFIT1, MX1, OAS3, OAS2, OAS1, IFIH1, IFITM1, CCL5, LAMP3, STAT1, ISG15, IFI27, EIF2AK2, TRIM21, OASL, IFITM3, BST2, IFITM2, IFI16, IFNB1, IRF7, TRIM22, PARP9, ADAR, APOBEC3G, TRIM5, TRIM25 |
| GO:0019079 | viral genome replication | 5.46E-24 | 20 | RSAD2, IFIT1, MX1, OAS3, OAS2, OAS1, IFIH1, IFITM1, CCL5, ISG15, IFI27, EIF2AK2, OASL, IFITM3, BST2, IFITM2, IFI16, IFNB1, ADAR, APOBEC3G |
| GO:0019058 | viral life cycle | 4.33E-23 | 25 | RSAD2, IFIT1, MX1, OAS3, OAS2, OAS1, IFIH1, IFITM1, CCL5, LAMP3, ISG15, IFI27, EIF2AK2, TRIM21, OASL, IFITM3, BST2, IFITM2, IFI16, IFNB1, TRIM22, ADAR, APOBEC3G, TRIM5, TRIM25 |
| GO:0045088 | regulation of innate immune response | 1.70E-22 | 22 | OAS3, OAS1, CCL5, ISG15, IFI35, TRIM21, IFI16, SAMHD1, LAG3, ZBP1, IFNB1, IRF7, PARP9, IRF1, DHX58, STAT2, ADAR, GBP5, HLA-B, TRIM5, AIM2, HLA-E |
| GO:0035456 | response to interferon-beta | 2.72E-19 | 12 | OAS1, IFITM1, XAF1, STAT1, IFITM3, BST2, IFITM2, IFI16, IFNB1, IRF1, TLR3, AIM2 |
| GO:0001819 | positive regulation of cytokine production | 5.15E-19 | 25 | RSAD2, OAS3, OAS2, DDX58, OAS1, IFIH1, STAT1, ISG15, IFNL1, EIF2AK2, IFI16, CD274, IRF7, IRF1, DHX58, GBP5, TLR3, CASP1, AIM2, JAK2, HLA-E, TLR2, IL15, MYD88, CXCL17 |
| GO:0034341 | response to interferon-gamma | 1.73E-18 | 17 | IFITM1, CCL5, GBP4, STAT1, SP100, TRIM21, IFITM3, BST2, GBP1, IFITM2, PARP9, IRF1, GBP5, TLR3, CASP1, JAK2, TLR2 |

# **Figures**


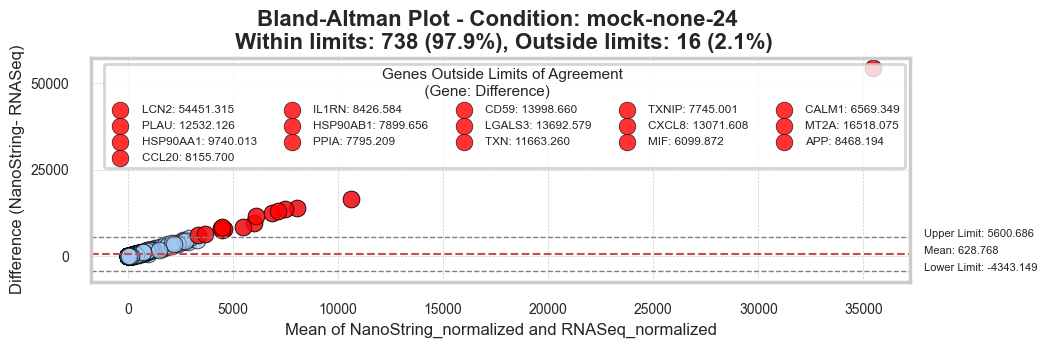

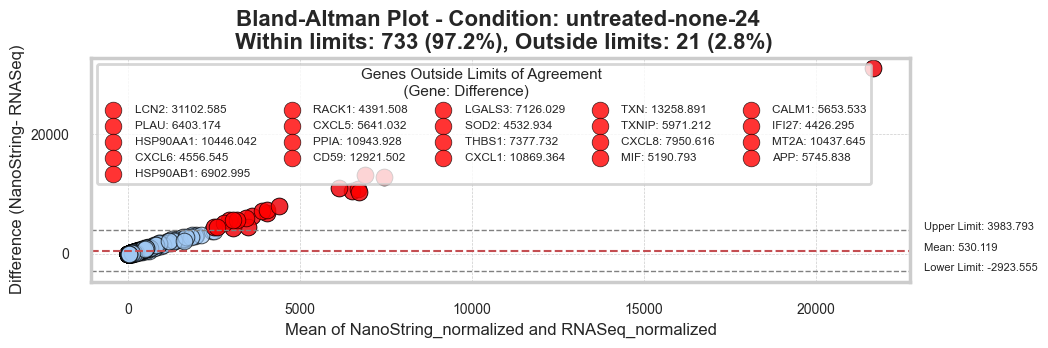

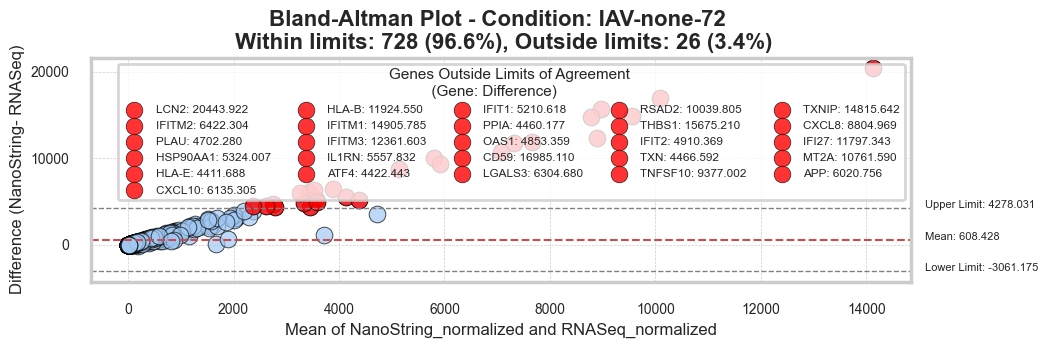


**Figure S1 (1).** Bland-Altman plots comparing gene expression measurements from RNA-Seq and NanoString platforms for each condition. The x-axis represents the average of the normalized counts from the two methods, while the y-axis represents the difference between them (NanoString - RNA-Seq). The dashed red line indicates the mean difference, while the dashed grey lines denote the limits of agreement (mean difference ± 1.96 standard deviations). Points representing individual genes are color-coded based on whether they are within or beyond the limits of agreement. Genes falling outside the limits are colored red and labeled with their difference values. The title of each plot indicates the condition, the total number of genes, and the proportion of genes within and outside the limits of agreement.


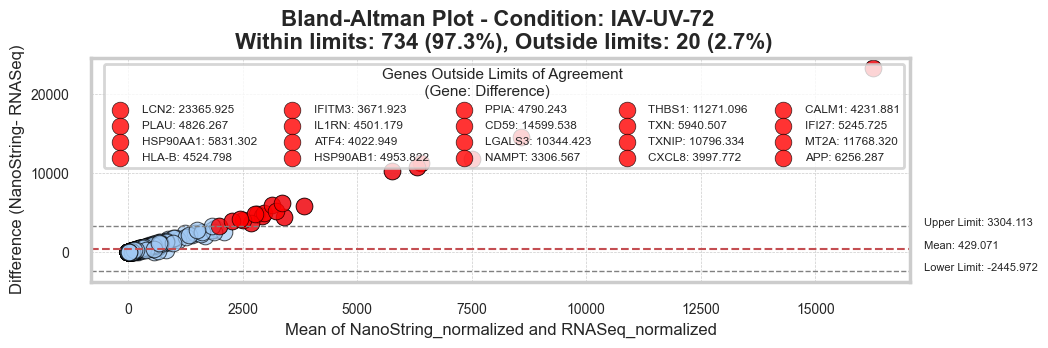

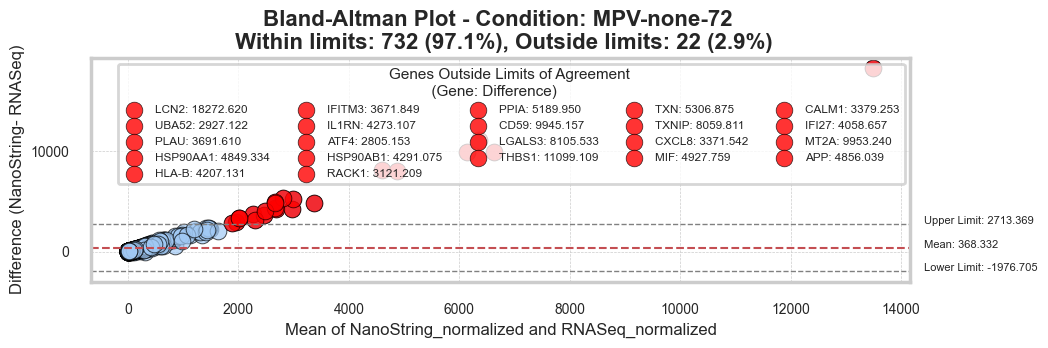

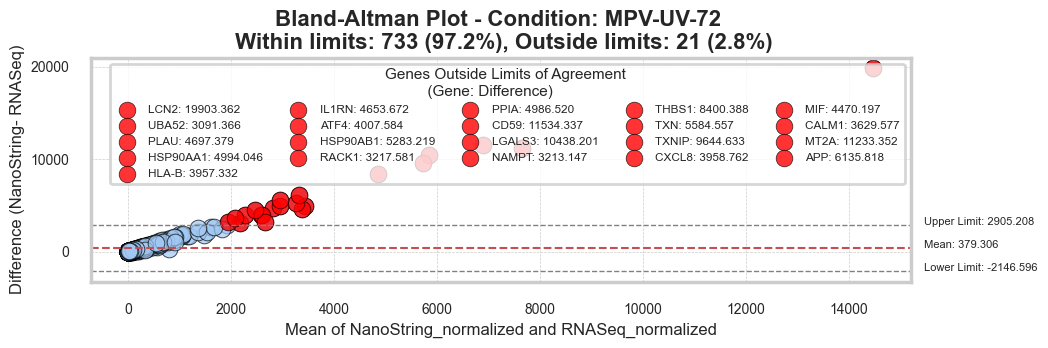


**Figure S1 (2).** Bland-Altman plots comparing gene expression measurements from RNA-Seq and NanoString platforms for each condition. The x-axis represents the average of the normalized counts from the two methods, while the y-axis represents the difference between them (NanoString - RNA-Seq). The dashed red line indicates the mean difference, while the dashed grey lines denote the limits of agreement (mean difference ± 1.96 standard deviations). Points representing individual genes are color-coded based on whether they are within or beyond the limits of agreement. Genes falling outside the limits are colored red and labeled with their difference values. The title of each plot indicates the condition, the total number of genes, and the proportion of genes within and outside the limits of agreement.


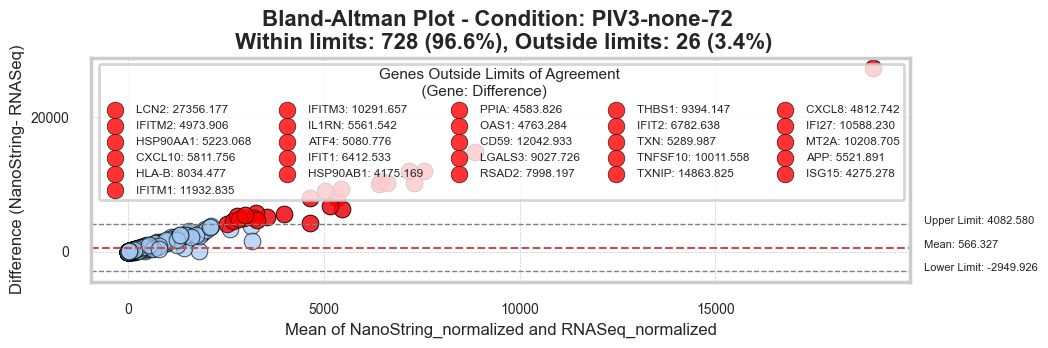

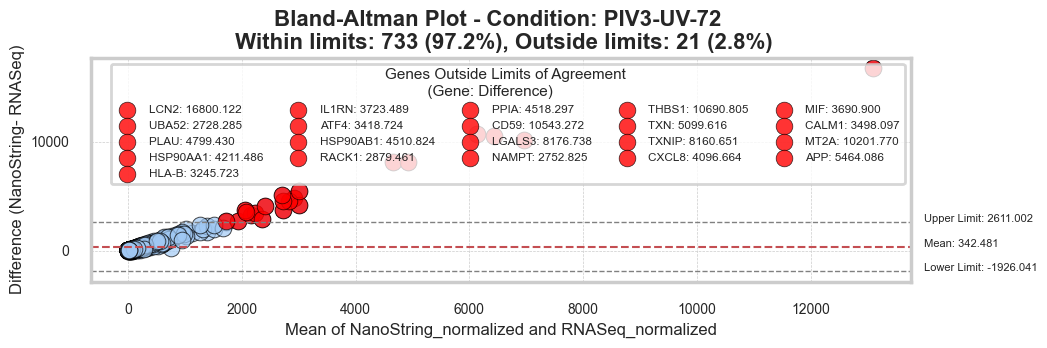

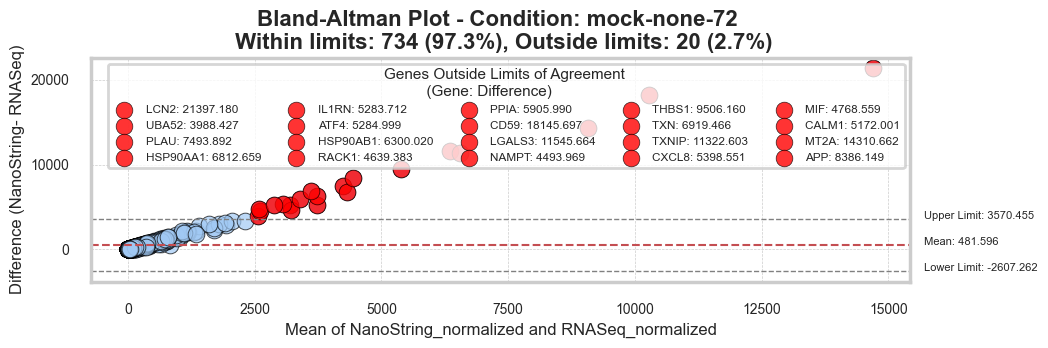


**Figure S1 (3).** Bland-Altman plots comparing gene expression measurements from RNA-Seq and NanoString platforms for each condition. The x-axis represents the average of the normalized counts from the two methods, while the y-axis represents the difference between them (NanoString - RNA-Seq). The dashed red line indicates the mean difference, while the dashed grey lines denote the limits of agreement (mean difference ± 1.96 standard deviations). Points representing individual genes are color-coded based on whether they are within or beyond the limits of agreement. Genes falling outside the limits are colored red and labeled with their difference values. The title of each plot indicates the condition, the total number of genes, and the proportion of genes within and outside the limits of agreement.


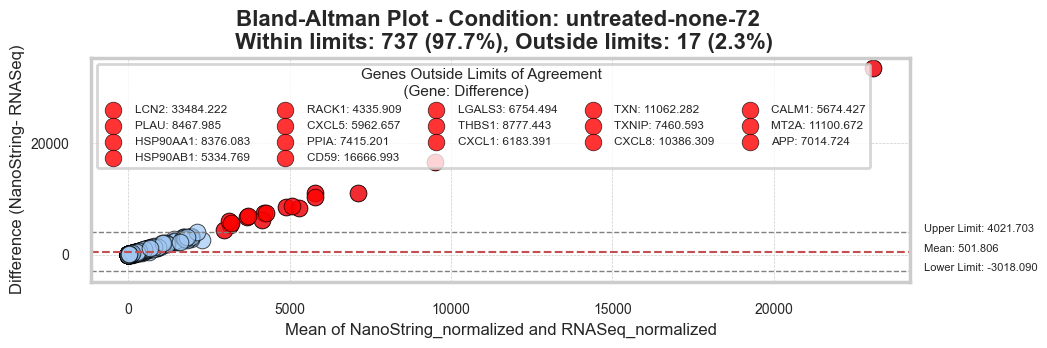


**Figure S1 (4).** Bland-Altman plots comparing gene expression measurements from RNA-Seq and NanoString platforms for each condition. The x-axis represents the average of the normalized counts from the two methods, while the y-axis represents the difference between them (NanoString - RNA-Seq). The dashed red line indicates the mean difference, while the dashed grey lines denote the limits of agreement (mean difference ± 1.96 standard deviations). Points representing individual genes are color-coded based on whether they are within or beyond the limits of agreement. Genes falling outside the limits are colored red and labeled with their difference values. The title of each plot indicates the condition, the total number of genes, and the proportion of genes within and outside the limits of agreement.

| 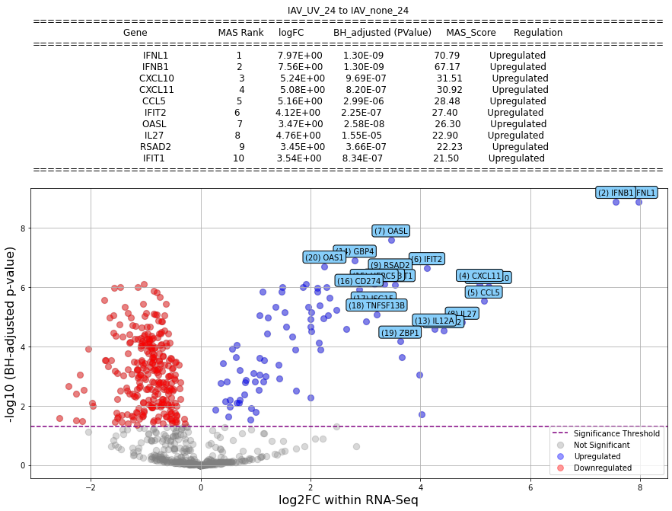 | 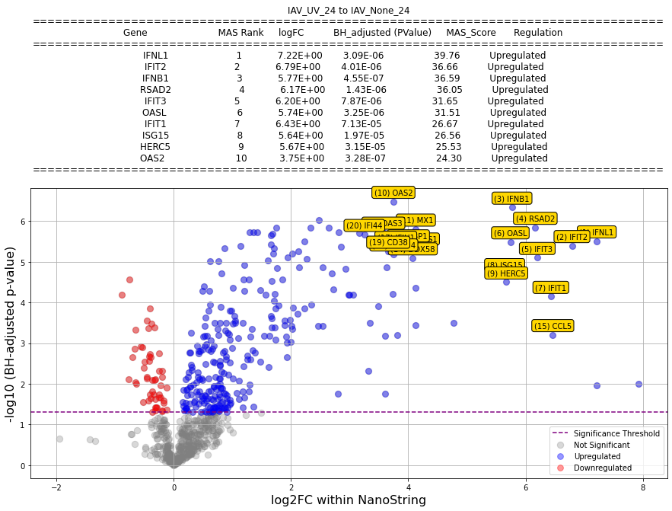 |
| --- | --- |
| 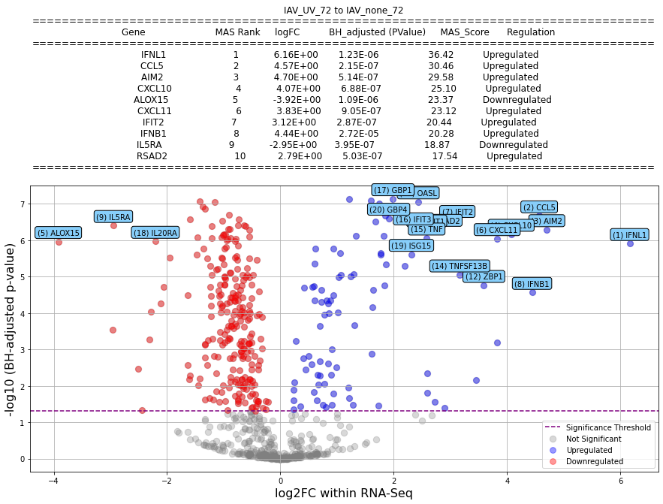 | 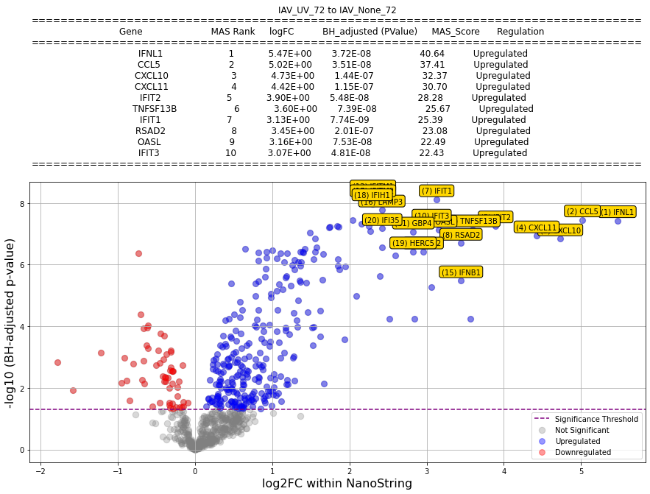 |
| 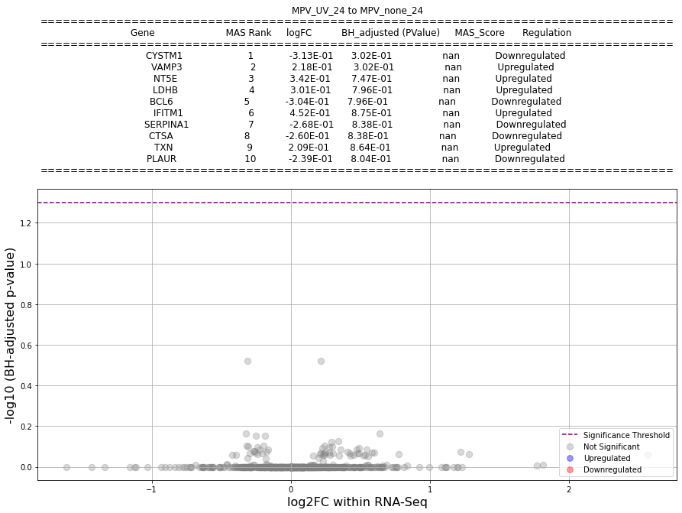 | 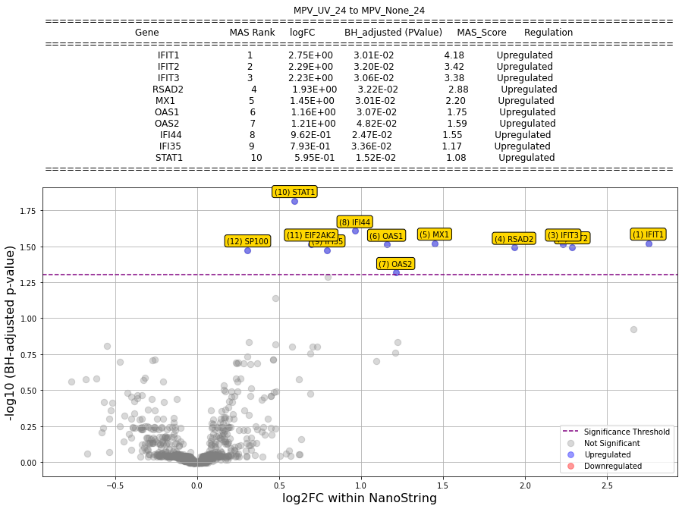 |

**Figure S2 (1).** Volcano plot comparing gene expression between Mock-24 and IAV-None-24 in RNA-seq and NanoString datasets.

| 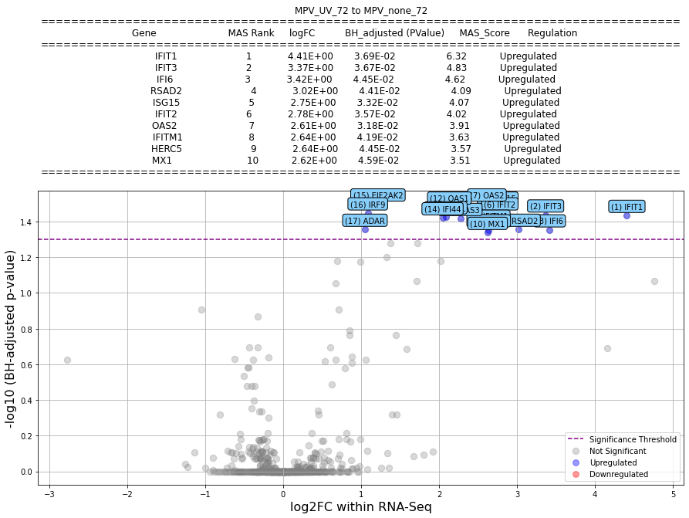 | 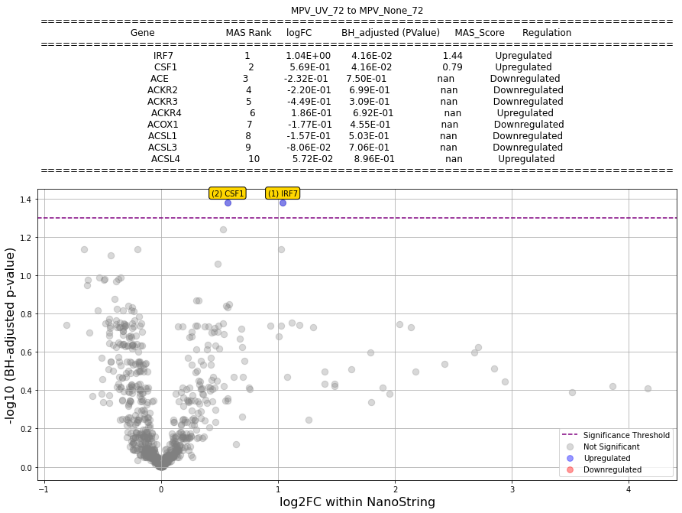 |
| --- | --- |
| 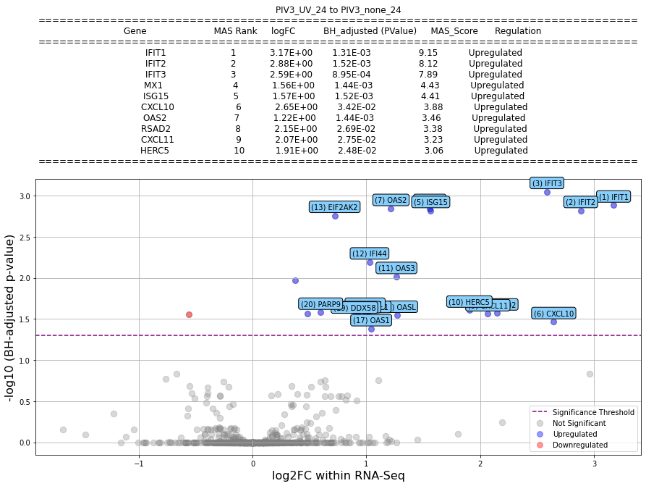 | 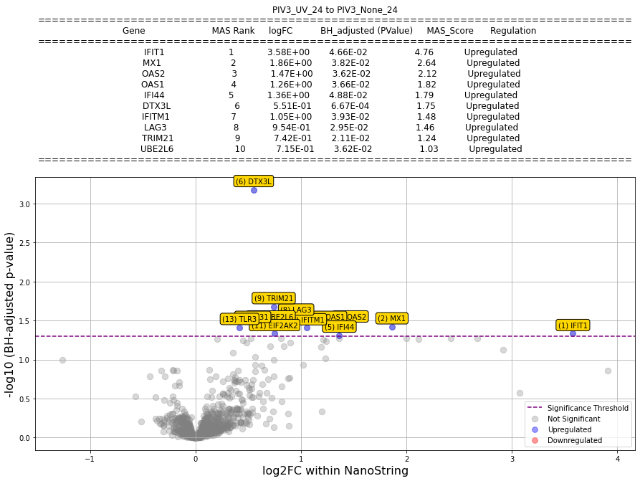 |
| 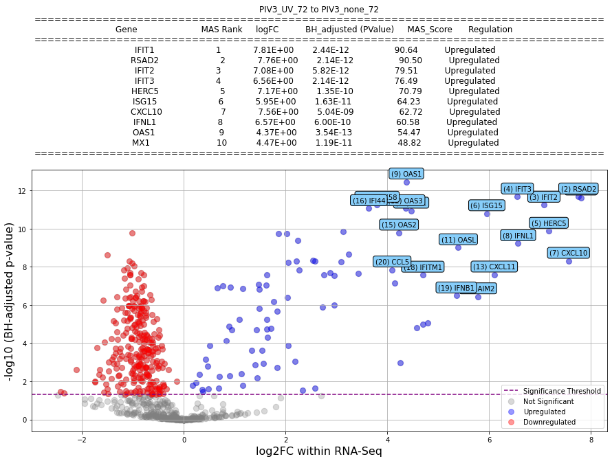 | 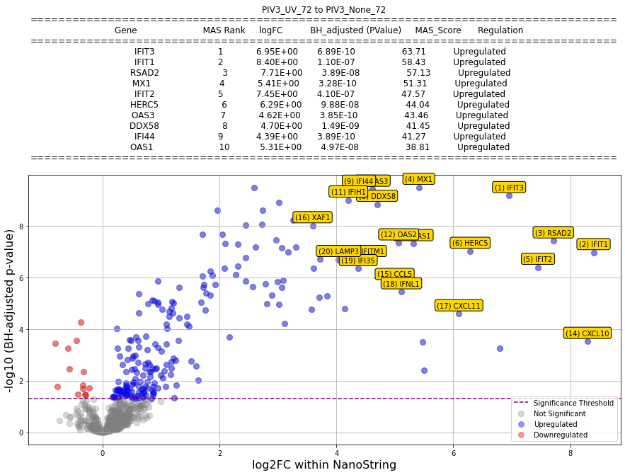 |

**Figure S2 (2).** Volcano plot comparing gene expression between Mock-24 and IAV-None-24 in RNA-seq and NanoString datasets.

| 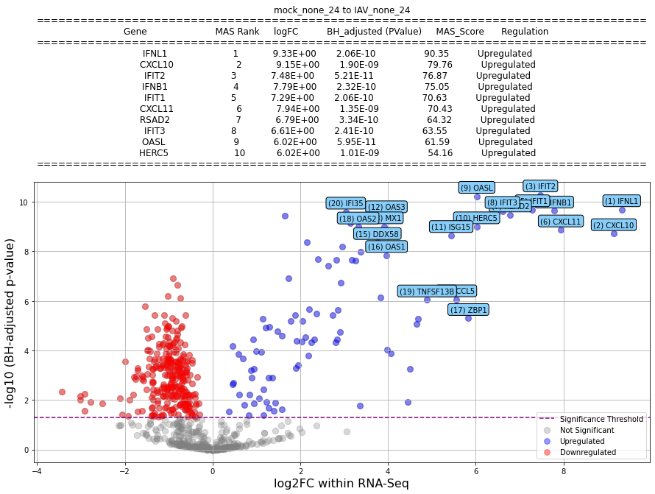 | 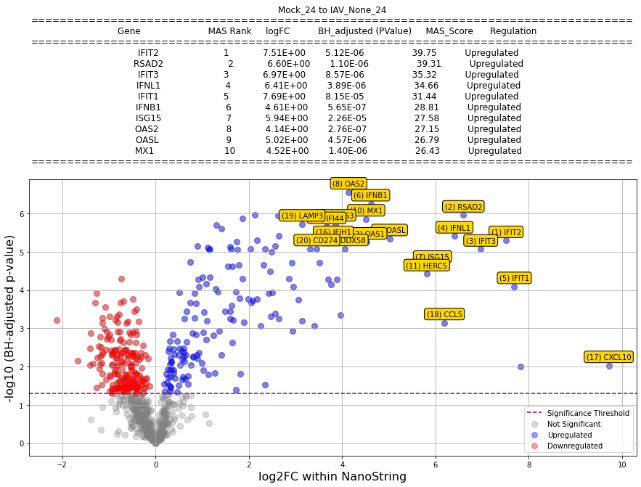 |
| --- | --- |
| 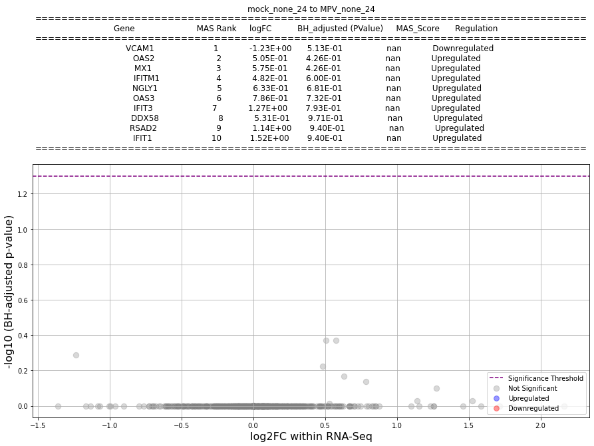 | 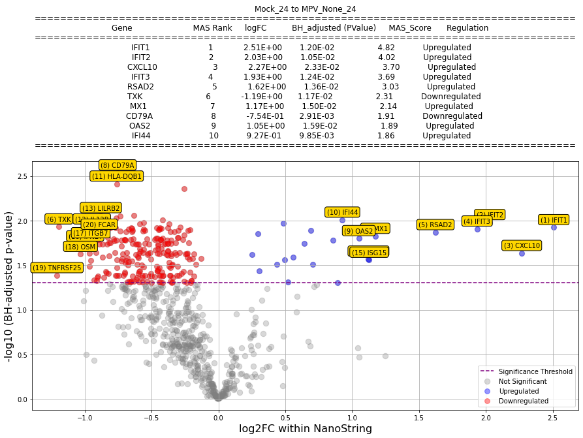 |
| 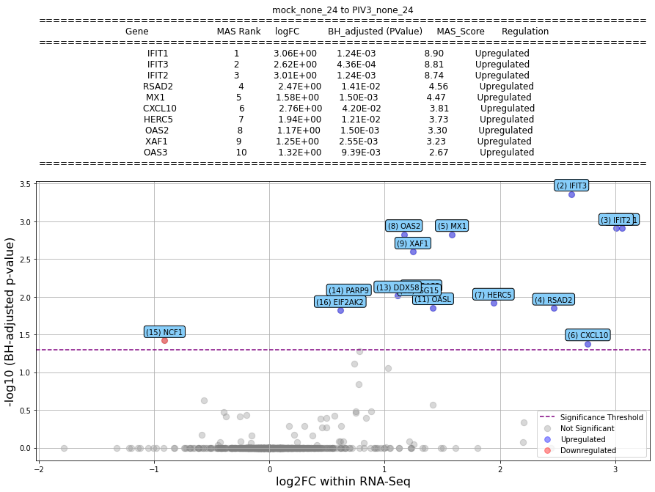 | 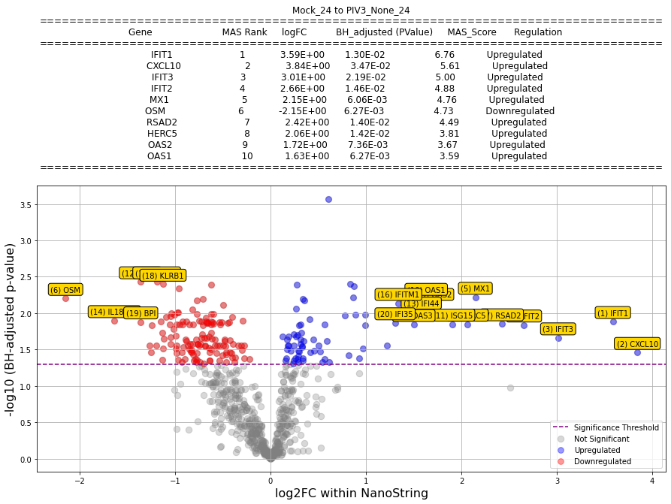 |

**Figure S2 (3).** Volcano plot comparing gene expression between Mock-24 and IAV-None-24 in RNA-seq and NanoString datasets.

| 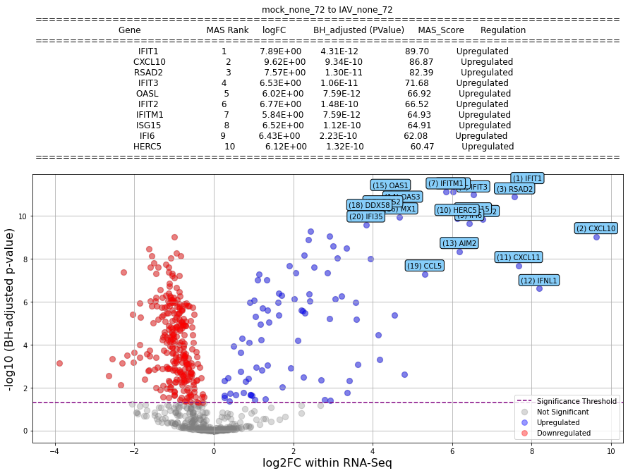 | 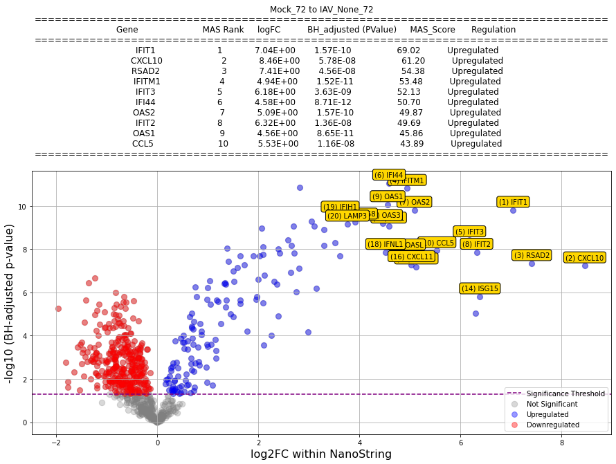 |
| --- | --- |
| 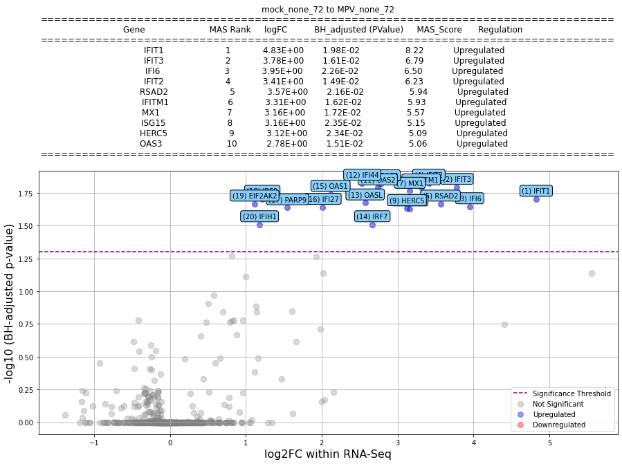 | 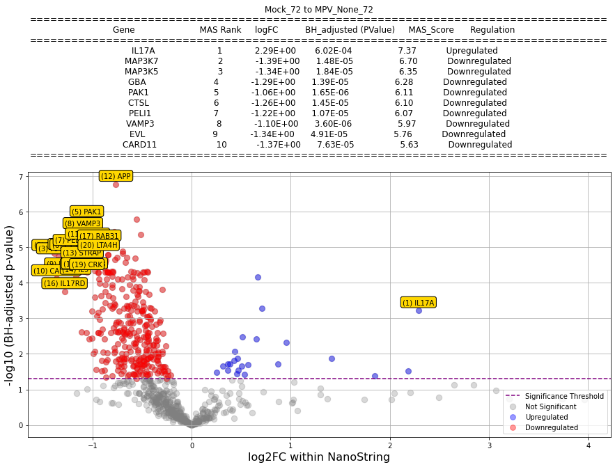 |
| 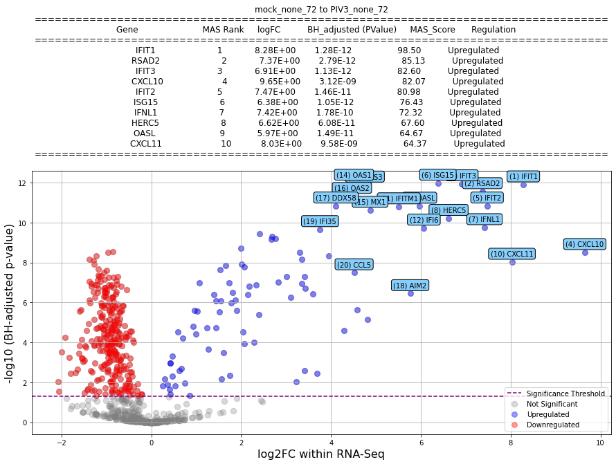 | 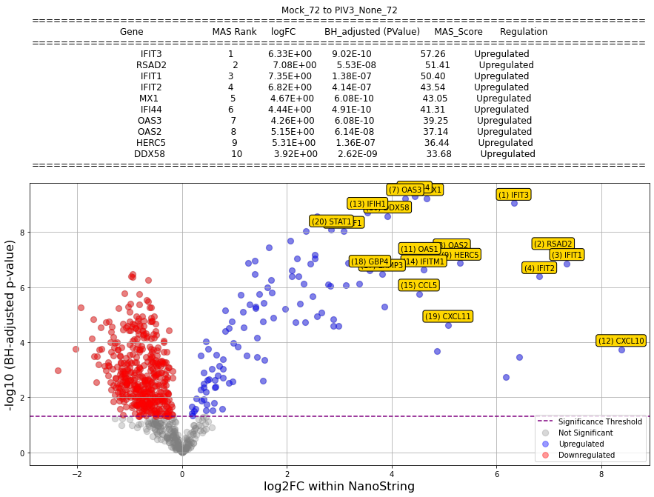 |

**Figure S2 (4).** Volcano plot comparing gene expression between Mock-24 and IAV-None-24 in RNA-seq and NanoString datasets.


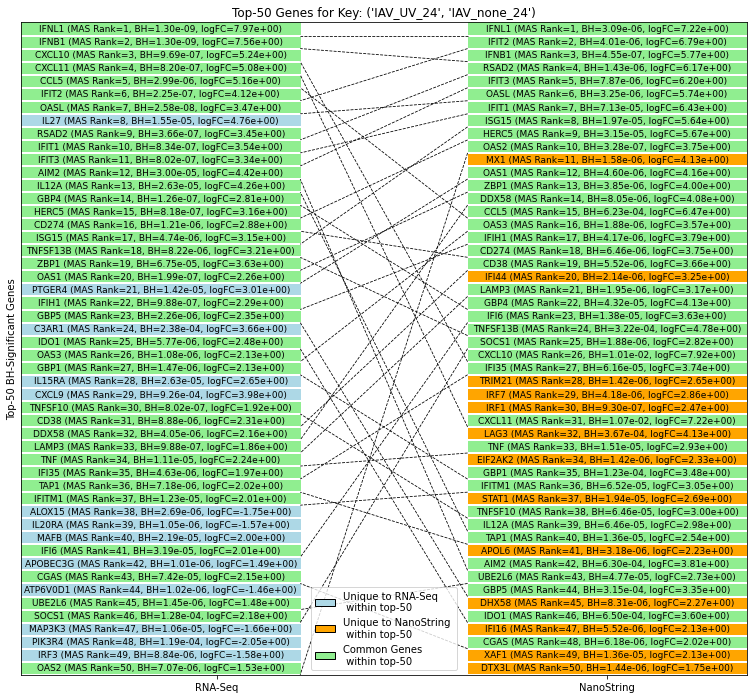


**Figure S3 (1).** Top 50 differentially expressed genes identified using the MAS algorithm, emphasizing the overlapping genes between RNA-Seq and NanoString platforms.


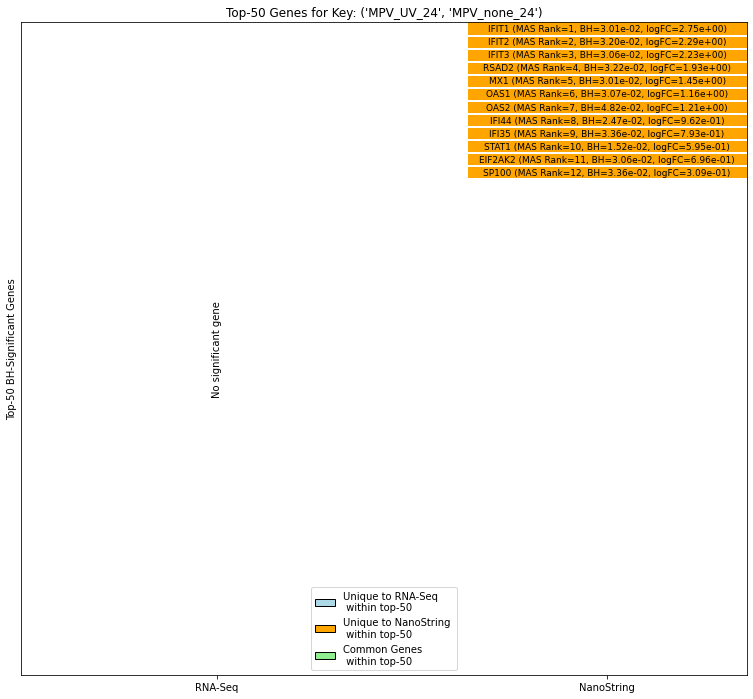


**Figure S3 (2).** Top 50 differentially expressed genes identified using the MAS algorithm, emphasizing the overlapping genes between RNA-Seq and NanoString platforms.


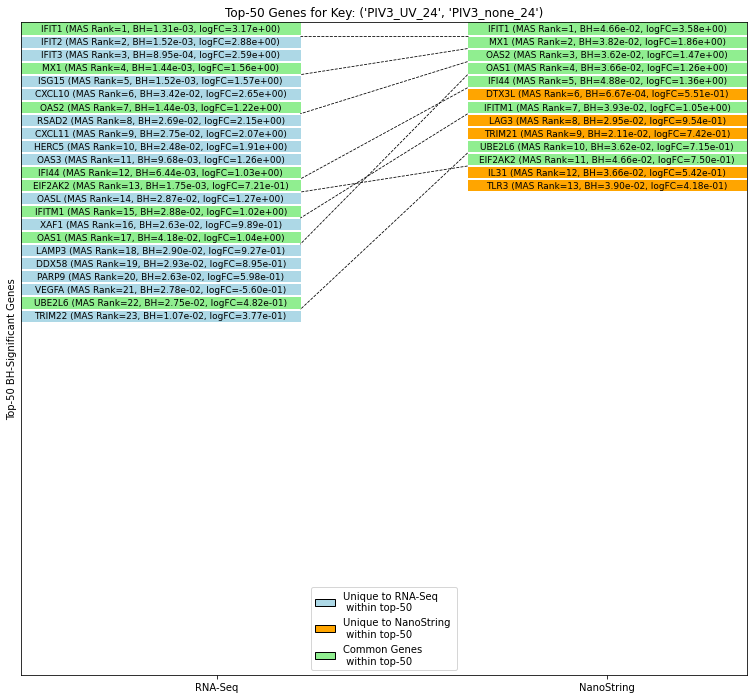


**Figure S3 (3).** Top 50 differentially expressed genes identified using the MAS algorithm, emphasizing the overlapping genes between RNA-Seq and NanoString platforms.


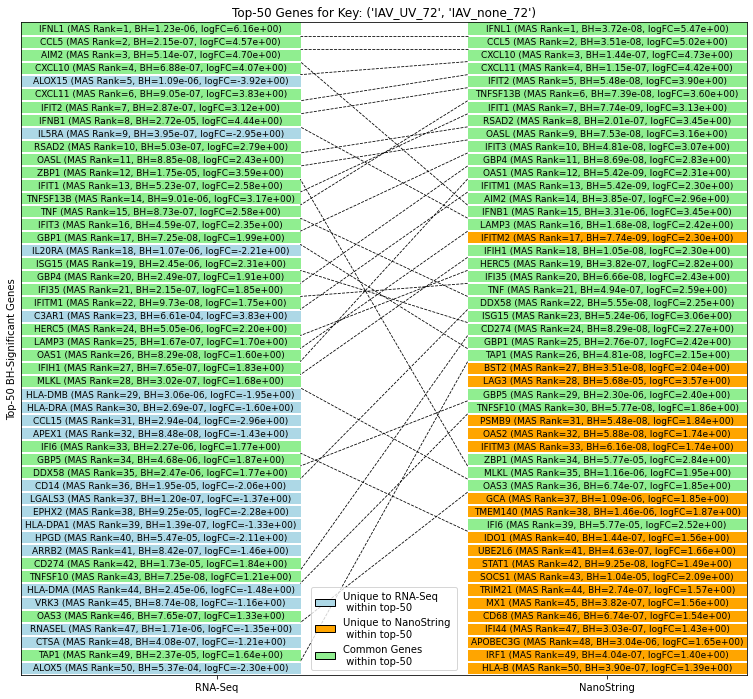


**Figure S3 (4).** Top 50 differentially expressed genes identified using the MAS algorithm, emphasizing the overlapping genes between RNA-Seq and NanoString platforms.


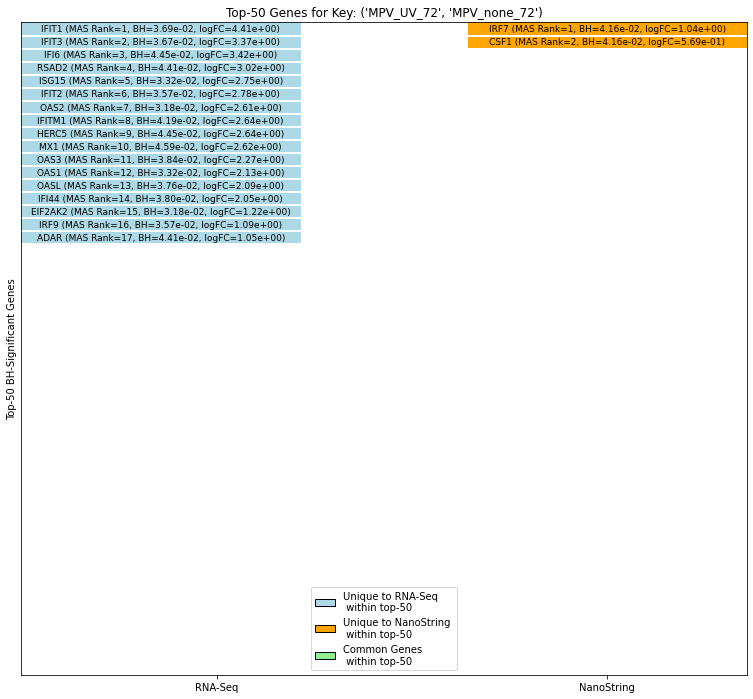


**Figure S3 (5).** Top 50 differentially expressed genes identified using the MAS algorithm, emphasizing the overlapping genes between RNA-Seq and NanoString platforms.


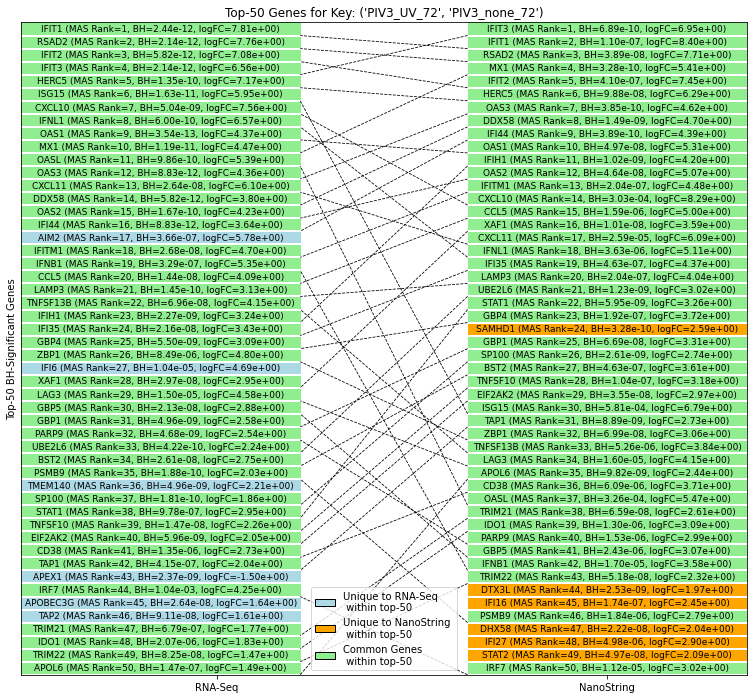


**Figure S3 (6).** Top 50 differentially expressed genes identified using the MAS algorithm, emphasizing the overlapping genes between RNA-Seq and NanoString platforms.


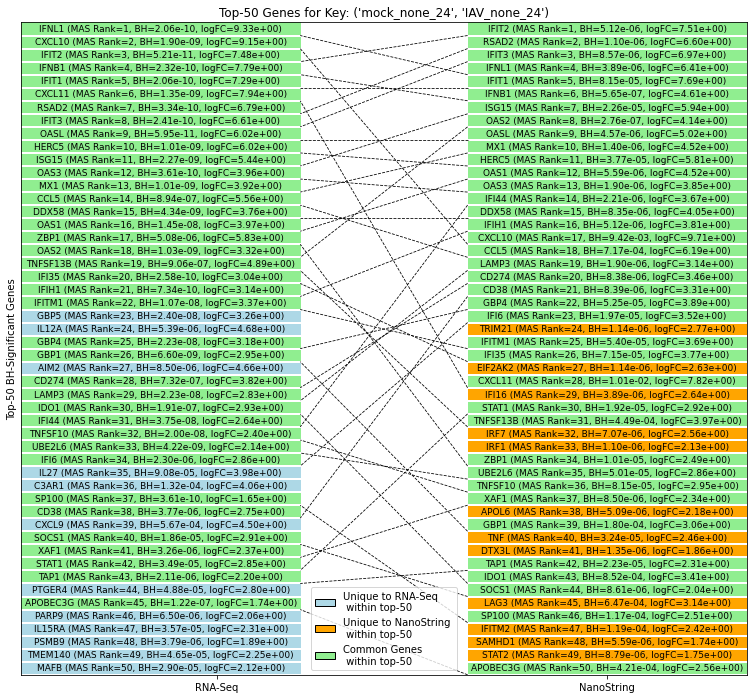


**Figure S3 (7).** Top 50 differentially expressed genes identified using the MAS algorithm, emphasizing the overlapping genes between RNA-Seq and NanoString platforms.


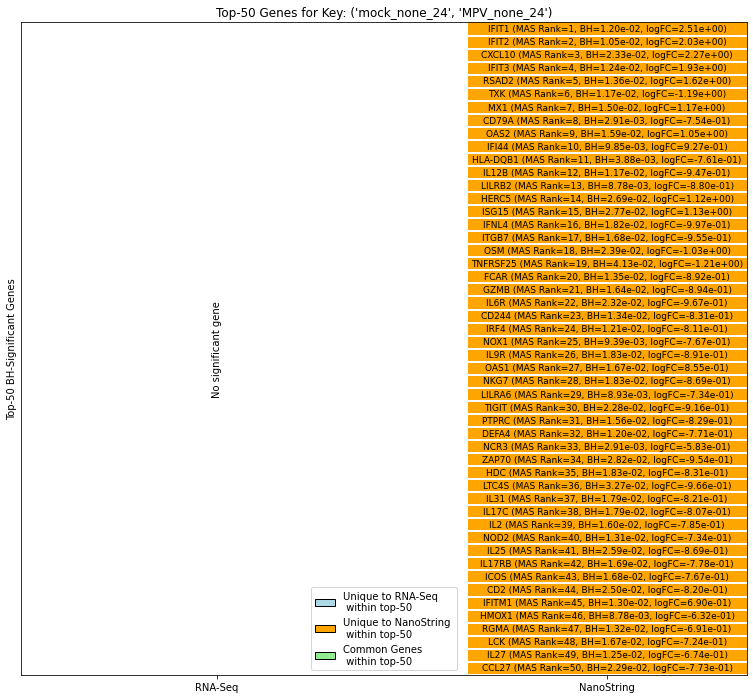


**Figure S3 (8).** Top 50 differentially expressed genes identified using the MAS algorithm, emphasizing the overlapping genes between RNA-Seq and NanoString platforms.


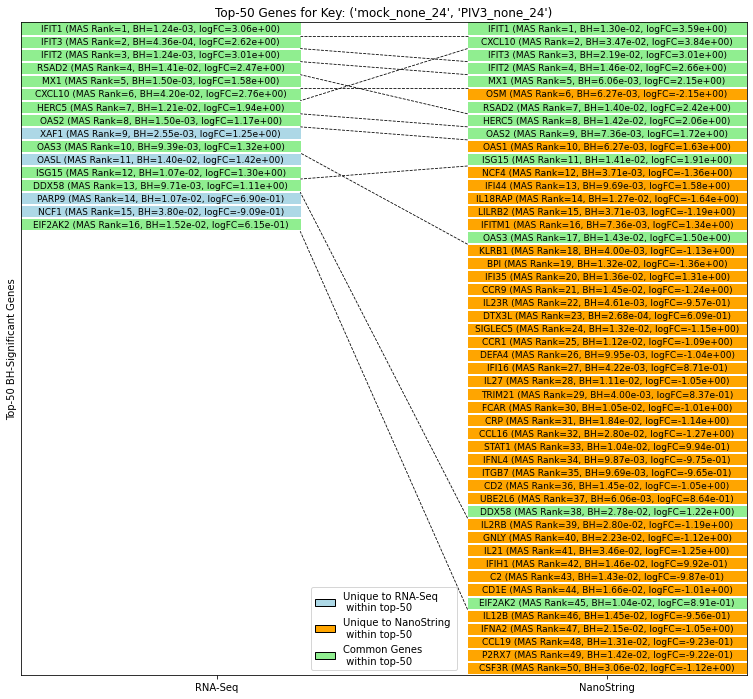


**Figure S3 (9).** Top 50 differentially expressed genes identified using the MAS algorithm, emphasizing the overlapping genes between RNA-Seq and NanoString platforms.


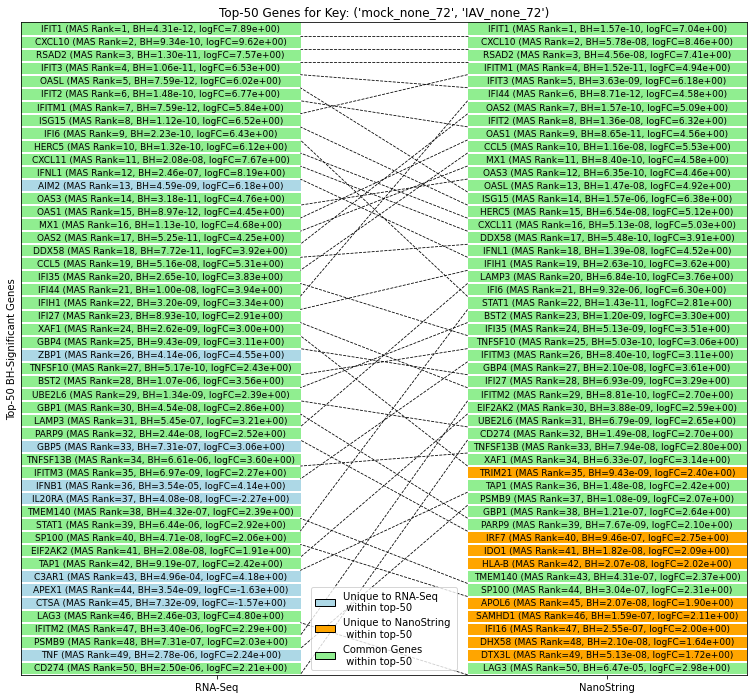


**Figure S3 (10).** Top 50 differentially expressed genes identified using the MAS algorithm, emphasizing the overlapping genes between RNA-Seq and NanoString platforms.


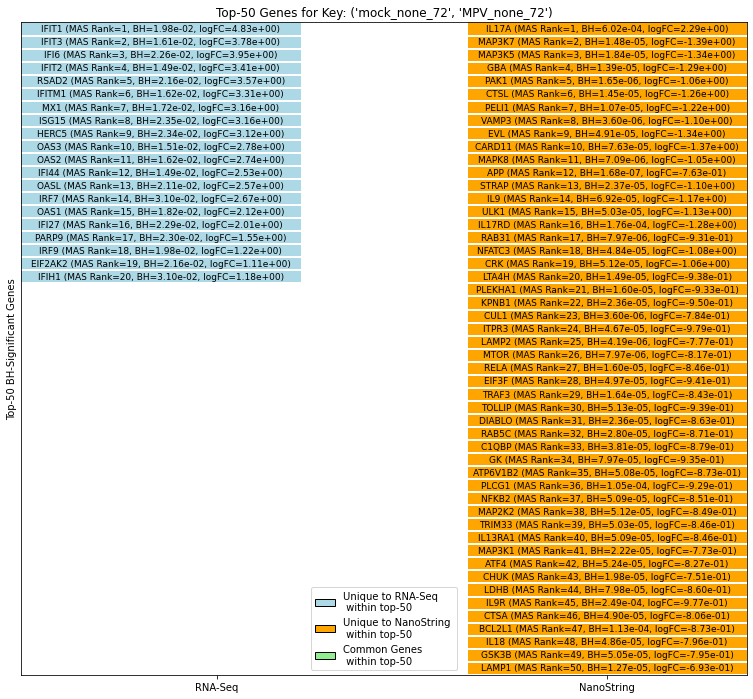


**Figure S3 (11).** Top 50 differentially expressed genes identified using the MAS algorithm, emphasizing the overlapping genes between RNA-Seq and NanoString platforms.


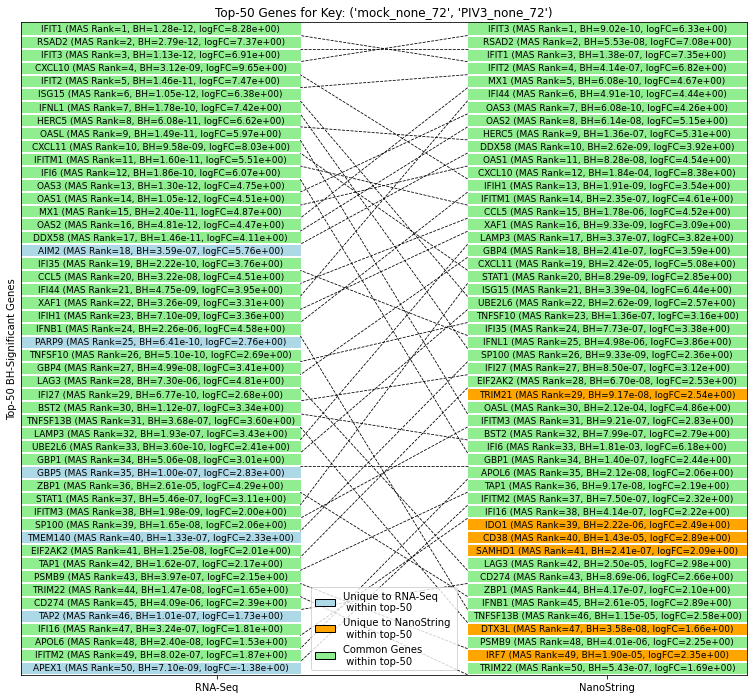


**Figure S3 (12).** Top 50 differentially expressed genes identified using the MAS algorithm, emphasizing the overlapping genes between RNA-Seq and NanoString platforms.
